# Vitamin K‐dependent gamma‐carboxyglutamic acid protein 1 promotes pancreatic ductal adenocarcinoma progression through stabilizing oncoprotein KRAS and tyrosine kinase receptor EGFR

**DOI:** 10.1002/ctm2.70191

**Published:** 2025-01-22

**Authors:** Zheng Wu, Qing Ye, Shan Zhang, Li‐Peng Hu, Xiao‐Qi Wang, Lin‐Li Yao, Lei Zhu, Shu‐Yu Xiao, Zong‐Hao Duan, Xue‐Li Zhang, Shu‐Heng Jiang, Zhi‐Gang Zhang, De‐Jun Liu, Dong‐Xue Li, Xiao‐Mei Yang

**Affiliations:** ^1^ State Key Laboratory of Systems Medicine for Cancer Shanghai Cancer Institute Ren Ji Hospital School of Medicine Shanghai Jiao Tong University Shanghai P.R. China; ^2^ Department of Biliary‐Pancreatic Surgery Ren Ji Hospital School of Medicine Shanghai Jiao Tong University Shanghai P.R. China

**Keywords:** KRAS, NEDD4 E3 ligases, pancreatic cancer, PRRG1, vitamin K‐dependent carboxylation

## Abstract

**Background:**

Vitamin K‐dependent γ‐glutamic acid carboxylation (Gla) proteins are calcium‐binding and membrane‐associated, participating in coagulation, bone turnover, and cancer biology. The molecular function of transmembrane proline‐rich Gla proteins (PRRGs) remains unexplored.

**Methods:**

Analysis of pancreatic ductal adenocarcinoma (PDAC) datasets, including transcription profiles, clinical data, and tissue microarrays, was conducted to evaluate PRRG1 expression and its clinical relevance. PDAC cell lines with overexpressed, knockdown, and mutated PRRG1 were developed to study biological functions and pathways using RNA‐seq, co‐immunoprecipitation with mass spectrometry, Western blotting, and immunofluorescence. In vivo xenograft and orthotopic models assessed PRRG1's impact on PDAC progression, with and without warfarin treatment.

**Results:**

PRRG1 was significantly upregulated in PDAC compared to normal pancreas, correlating with poorer patient survival. PRRG1 knockdown reduced PDAC cell proliferation, anchorage‐independent growth in vitro, and tumor growth in vivo. PRRG1 localized at the plasma membrane, interacted with the HECT E3 ligase NEDD4 via the C‐terminal PPXY motif, and promoted NEDD4 self‐ubiquitination, reducing its protein levels. PRRG1 knockdown elevated NEDD4, destabilizing the oncoprotein KRAS and receptor EGFR, and attenuating downstream signaling and macropinocytosis under nutrient deprivation. The vitamin K‐dependent Gla modification of PRRG1 was crucial for its membrane localization and pro‐tumorigenic effects, and was inhibited by low‐dose warfarin, a clinical vitamin K antagonist.

**Conclusions:**

This study identifies PRRG1 as a key regulator of pro‐tumorigenic signaling in PDAC, suggesting the potential of repurposing the anticoagulant warfarin as a therapeutic strategy.

**Key points:**

- PRRG1 is identified as the transmembrane Gla protein mediating PDAC malignancy.
- PRRG1 recruits and induces self‐ubiquitination of membrane‐anchoring E3 ligase NEDD4.
- PRRG1 exerts a protective role toward KRAS and EGFR by inhibiting NEDD4.
- The anticoagulant warfarin can be utilized to inhibit PRRG1 and PDAC advancement.

## Graphical abstract

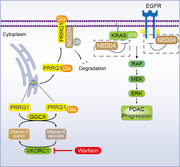

## 1 INTRODUCTION

Pancreatic cancer, though relatively uncommon, presents a dire prognosis, with nearly as many deaths (466 000) as cases (496 000) reported in 2020.^1^ Constituting the predominant form (90%) of pancreatic neoplasms, pancreatic ductal adenocarcinoma (PDAC) is distinguished by numerous genetic mutations and intricate tumour microenvironments, resulting in formidable resistance to conventional treatment modalities.^2^, ^3^ Therefore, research aimed at unravelling the molecular mechanisms behind PDAC can pave the way for targeted therapies that may offer better outcomes for patients.

Acquisition of oncogenic mutations, primarily the activating mutations in the oncogene KRAS, initiates and coordinates the development of PDAC.^4^ The PDAC is featured with active stroma and poorly‐formed vascular systems, constituting a nutrient‐limiting and immune‐suppression microenvironment.^5^ Active mutant KRAS drives critical signalling cascades for tumour growth, endocytosis and cytoskeletal organization.^6^ Particularly, oncogenic RAS‐expressing cells activate macropinocytosis, a non‐selective endocytic process, to internalize and uptake nutrients from the microenvironment.^7^, ^8^ KRAS is anchored to the inner membrane of the cell for proper signal transduction following post‐translational lipid modification. The average retention time of KRAS on the plasma membrane is about 8 min and its stability involves the ubiquitination and endocytosis processes.^9^, ^10^

As a vital post‐translational modification in the human body, vitamin K‐dependent carboxylation is responsible for converting designated glutamate residues in vitamin K‐dependent proteins into gamma‐carboxyglutamate (Gla) residues.^11^ Catalyzed by a special enzyme gamma‐glutamyl carboxylase (GGCX), this process depends on vitamin K hydroquinone, oxygen, and carbon dioxide as essential cofactors.^12^ The production of reduced vitamin K, essential for this reaction, is mediated by vitamin K oxidoreductase C1 (VKORC1), which can be inhibited by warfarin. Warfarin exerts its anticoagulant effect by inhibiting VKORC1, which decreases the availability of active vitamin K (KH2). This reduction in active vitamin K disrupts the carboxylation of vitamin K‐dependent clotting factors, thereby preventing blood clot formation.^13^ Therefore, vitamin K‐dependent carboxylation is indispensable for the biological activity of vitamin K‐dependent proteins, especially clotting factors (such as factors II, VII, IX, and X) and anticoagulants (such as protein C, protein S, and protein Z).^14^ For instance, the clustered Gla domains within coagulation factors undergo a structural transition upon calcium binding, exposing a phospholipid binding site crucial for membrane attachment and clot formation.^15^ Other Gla proteins, such as matrix Gla protein, osteocalcin, growth arrest‐specific 6 (GAS6), and Gla‐rich protein, play diverse roles in physiological and pathological processes, including arterial calcification prevention, bone turnover, body weight and glucose regulation, corneal health, sperm maturation, and cancer progression.^16^ Specifically, the γ‐carboxylation of GAS6 was implicated in the activation of AXL receptor tyrosine kinase in aggressive breast cancer and PDAC, which can be impeded by low‐dose warfarin independent of anticoagulation.^17^, ^18^

By analyzing expression profiles from the Gene Expression Omnibus (GEO) and The Cancer Genome Atlas (TCGA) databases, we found that proline‐rich and Gla domain 1 (*PRRG1*), a member of the PRRG family, is linked to poor prognosis in PDAC patients with overexpression. Located on chromosome X, this gene encodes a Vitamin K‐dependent gamma‐carboxylated, single‐pass transmembrane protein.^19^ PRRG proteins are predicted to span approximately 200 amino acids, featuring Gla residues on the extracellular side, and in the cytosolic domain containing two consecutive Pro/Leu‐Pro‐Xaa‐Tyr (PPXY) motif, which serves as the minimal structure bound by WW domains (type I).^20^ Despite the broad tissue expression of this family, their molecular functions were not clear. Limited literature indicated proteins containing SH3 and WW motifs as potential cytosolic binding partners for PRRG2 and PRRG4.^21^, ^22^ To date, the possible functions and mechanisms of PRRGs have not been elucidated.

In this study, we unveiled a significant link between the elevated PRRG1 and the malignancy of PDAC, shedding light on the regulatory role of PRRG1 on the maintenance of protein stabilities of KRAS and EGFR through negative regulation of the E3 ligase NEDD4. PRRG1 requires vitamin K‐dependent γ‐glutamic acid carboxylation for proper positioning and functioning, which can be targeted with the clinical anticoagulant warfarin.

## 2 RESULTS

### 2.1 PRRG1 is upregulated in pancreatic ductal adenocarcinoma and associated with patients’ survival

**FIGURE 1:**
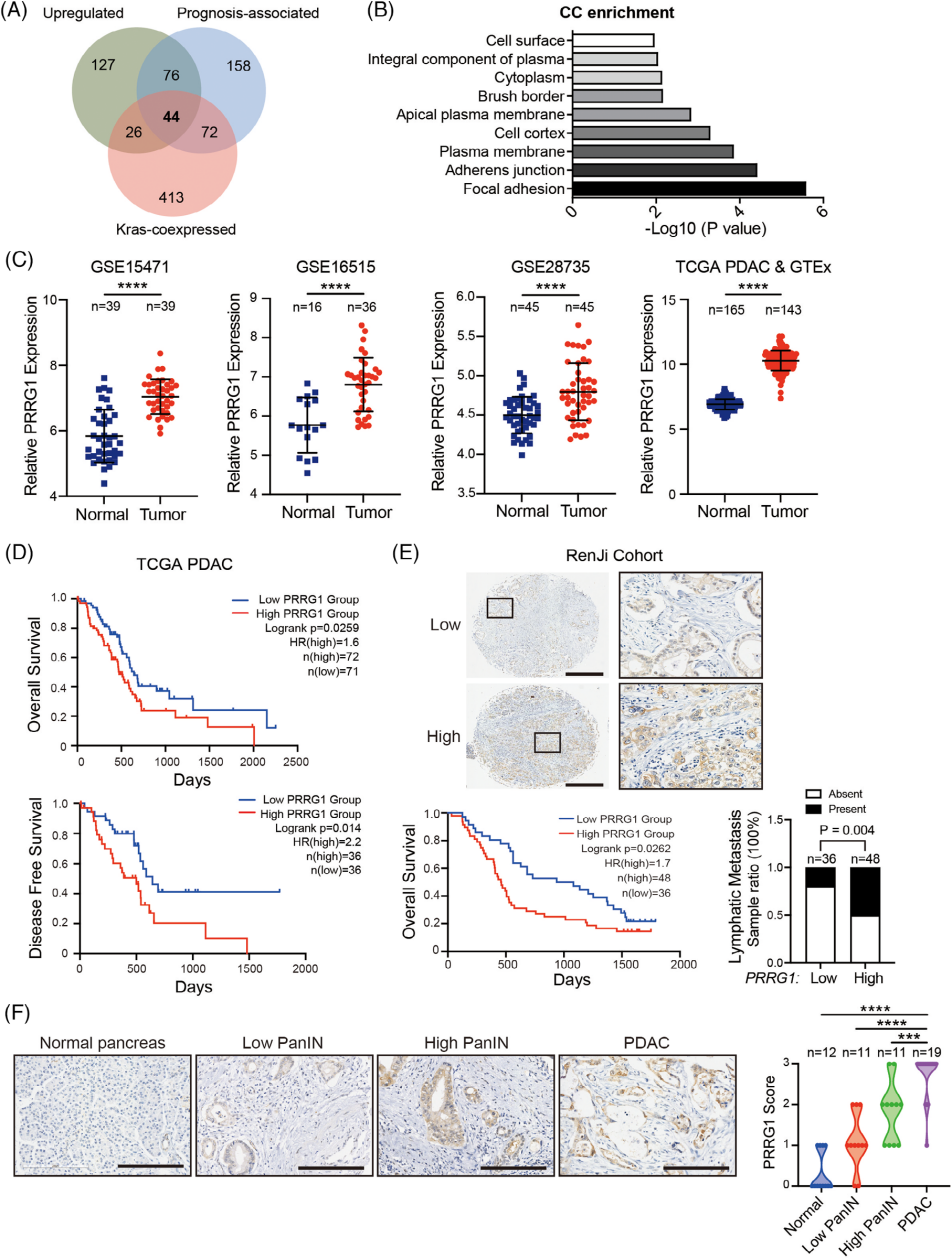
Expression profiles showing PRRG1 upregulation in cancer and its correlation with patient prognosis. (A) Venn diagram showing 44 shared genes obtained from three different gene sets, including a TCGA PAAD prognosis‐associated gene set, a TCGA PAAD KRAS‐coexpressed gene set and a gene set with upregulated genes in three GEO datasets (GSE15471, GSE16515 and GSE28735). (B) Gene ontology analyses of the cellular component (CC) of the 44 shared genes in (A) using the Database for Annotation, Visualization and Integrated Discovery (DAVID) functional annotation chart tool. (C) PRRG1 mRNA level in PDAC cancer tissues versus normal tissues. Data were analyzed from GEO datasets, TCGA PDAC, and GTEx. (D) Kaplan–Meier plot of the overall survival and disease‐free survival of PDAC patients based on the expression of PRRG1, using TCGA PDAC databases. (E) Representative immunohistochemical images of high or low PRRG1 expression and its regional magnification in PDAC tissues (upper) and Kaplan–Meier survival analysis of patients with strong or weak PRRG1 expression (lower left), using TMA1 from Renji hospital (Renji cohort). Scale bar: 500 µm. The correlation of PRRG1 expression with lymphatic metastasis in the Renji TMA1 cohort was shown in the lower‐right panel. (F) Representative immunohistochemical images of PRRG1 staining in normal pancreas, low PanIN, high PanIN and PDAC tissues. The statistical analysis results of PRRG1 scores in the normal pancreas (*n* = 12), low‐grade PanIN (*n* = 11), high‐grade PanIN (*n* = 11), and PDAC lesions (*n* = 19) are shown on the right. Scale bar: 200 µm. The statistical tests used were: gene expression analysis, unpaired *t*‐test; patient survival, log‐rank test; correlation of PRRG1 expression with lymphatic metastasis, Pearson χ^2^ (two‐sided). The statistical significance of differences among different stages of pancreatic lesions was calculated by one‐way ANOVA with Dunnett's post hoc test. ***p < .001. ****p < .0001. GEO, Gene Expression Omnibus; GSE, Genomic Spatial Event database; TCGA, The Cancer Genome Atlas Program; GTEx, Genotype‐Tissue Expression Program; PAAD, pancreatic adenocarcinoma; PanIN, pancreatic intraepithelial neoplasia; PDAC, pancreatic ductal adenocarcinoma.

To discover key biological processes associated with PDAC malignancy, we utilized the TCGA Pancreatic Adenocarcinoma Database (TCGA‐PAAD) for analysis. Kyoto Encyclopedia of Genes and Genomes (KEGG) pathway enrichment revealed that the top 350 genes positively related to PDAC prognosis were remarkably enriched in cell plasma membrane‐associated events, including cell adhesion, endocytosis and the PI3K‐AKT pathway (Figure S1A). Considering the dominant role of mutant KRAS in PDAC carcinogenesis, these prognosis‐related genes were further intersected with 555 KRAS‐coexpressed genes in TCGA‐PAAD (Spearman's Correlation >.45, 168 samples, pancancer atlas) and 273 commonly upregulated genes in all three GEO malignant pancreatic cancer datasets with paired cancer and non‐cancerous tissues (GSE15471, GSE16515, and GSE28735; FC > 1). As a result, 44 shared genes were obtained (Figure 1A) and these genes mostly encode proteins that were associated with the plasma membrane and engaged in integrin signals (e.g., ITGA2, ITGB6, LAMA3), transmembrane receptor signals (MET, EPS8, PRKCI, GPRC5A, EFNB2) or metal endopeptidase activities (ADAM9, ADAM10; Figure 1B; Table S1). The above analysis revealed that membrane‐associated events and signalling played crucial roles in PDAC malignancy. Therefore, we focused on those enriched in the integral component of the plasma membrane, containing EFNB2, GPRC5A, PERP, PRRG1, SLC39A10, SGMS2, IL1RAP, MET, and SLC5A3. Amongst, the molecular function of a predicted vitamin K‐dependent Gla protein, the single‐pass transmembrane protein PRRG1, remains undetermined.

To investigate the expression profiles of PRRG1 in pancreatic cancer, we analyzed GEO datasets containing paired cancer and non‐cancerous specimens. PRRG1 was significantly upregulated in all three GEO datasets (GSE15471, GSE16515, and GSE28735). Besides, PRRG1 was evidently upregulated in TCGA PAAD compared with normal pancreas from the Genotype‐Tissue Expression (GTEx) databases (Figure S1B). PDAC samples, the main sample type in the TCGA PAAD dataset, were singled out for analysis and similar results were obtained (Figure 1C). In TCGA cohorts of PDAC and PAAD patients, increased PRRG1 mRNA expression was correlated with reduced overall survival and disease‐free survival (Figure 1D, Figure S1C). Next, we further examined the protein level of PRRG1 by immunohistochemical staining, using a PDAC tissue microarray from the Renji cohort containing 84 PDAC samples. Predominant immunostaining of PRRG1 was detected and observed on the plasma membrane of cancer cells, with high PRRG1 expression positively correlated with poor survival (Figure 1E). Further analysis demonstrated a strong correlation between PRRG1 expression and lymphatic metastasis. (Figure 1E; Table S2).

Moreover, we found that PRRG1 was nearly undetectable in normal pancreatic acinar cells or ductal cells while it was gradually upregulated in precursor lesions (pancreatic intraepithelial neoplasia, PanINs) and PDAC cancer cells (Figure 1F). These findings indicated that PRRG1 is upregulated in PDAC and is associated with PDAC malignant progression.

### 2.2 PRRG1 knockdown suppresses the malignant behaviours of PDAC cells

**FIGURE 2:**
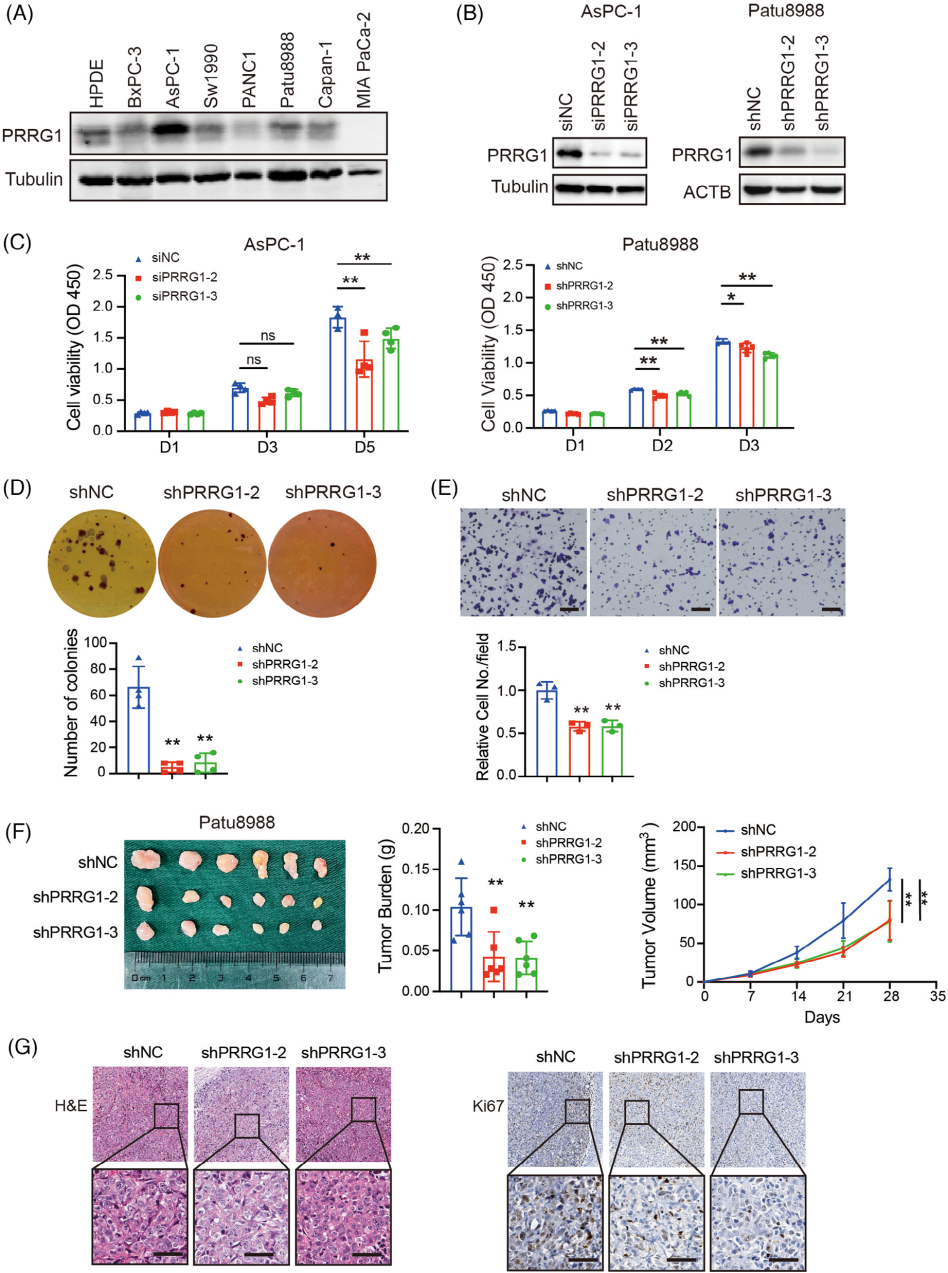
PRRG1 knockdown suppresses PDAC cancer growth in vitro and in vivo. (A) Western blot analysis of PRRG1 protein expression in PDAC cells. (B) The siRNA‐mediated PRRG1‐knockdown efficiency in AsPC‐1 cells and shRNA‐mediated PRRG1 knockdown efficiency in Patu8988 cells were analyzed by Western blotting. (C) Cell proliferation of PRRG1‐knockdown and control PDAC cells. The statistical significance of differences among multiple groups was calculated by two‐way ANOVA with Bonferroni's post hoc tests (*n* = 4 for each group). (D) Anchorage‐independent colony formation in PRRG1‐knockdown and control cells. The number of colonies formed per well was analyzed using one‐way ANOVA with Dunnett's post hoc test (*n* = 3 for each group). (E) Analysis of cell migration by transwell assay in PRRG1‐knockdown and control cells. The relative cell number per field was analyzed using one‐way ANOVA with Dunnett's post hoc test (*n* = 3 for each group). Scale bar: 100 µm. (F) Subcutaneous tumour growth in mice inoculated with shNC, shPRRG1‐2, or shPRRG1‐3 Patu8988 cells (*n* = 6 for each group). The results of the statistical analysis of tumour weight and tumour growth curve are shown on the right. (G) Representative images of hematoxylin and eosin (H&E) and immunohistochemical staining of Ki‐67 in tumours. Scale bar: 50 µm. The plotted values were presented as the mean ± SD. The statistical significance of differences among multiple groups was calculated by one‐way ANOVA with Dunnett's post hoc test or two‐way ANOVA with Bonferroni's post hoc tests. *p < .05, **p < .01. si, small interfering; sh, short hairpin. Images in (A) and (B) are representative of two independent experiments.

Western blotting was used to detect PRRG1 expression in the immortalized human pancreatic ductal epithelial cell line and a majority of PDAC cell lines (Figure 2A). To explore the function of PRRG1 in PDAC progression, small interfering RNAs (siRNAs) or lentiviral shRNA plasmids targeting *PRRG1* were introduced to PDAC cells for transient or stable knockdown of PRRG1 (Figure 2B). The results demonstrated that PRRG1 knockdown significantly suppressed the in vitro proliferation and anchorage‐independent growth of PDAC cells (Figure 2C,D). Following serum‐free starvation treatment, PRRG1 knockdown further increased apoptosis in PDAC cells, as evidenced by Annexin V/PI staining with flow cytometry (Figure S2A). Additionally, we found that PRRG1 knockdown also inhibited PDAC cell migration through the transwell polycarbonate membrane (Figure 2E).

To evaluate the impact of PRRG1 on PDAC growth in vivo, Patu8988 cells expressing lentiviral shRNA targeting PRRG1 or scrambled control cells were subcutaneously inoculated to the female M‐NSG immunodeficient mice of 6‐week‐old. After 4 weeks of growth, the tumours were dissected and measured. The result demonstrated that the in vivo tumour growth of PDAC cells was evidently suppressed in PRRG1‐knockdown groups compared with the control group (Figure 2F). Immunohistochemical staining and analysis showed that shPRRG1 tumours exhibited less proliferating and more apoptotic cells, as defined by Ki67‐ and TUNEL‐positive cell ratio, respectively (Figure 2G; Figure S2B).

### 2.3 Gla modification is indispensable for PRRG1 membranous localization and protein stability

**FIGURE 3:**
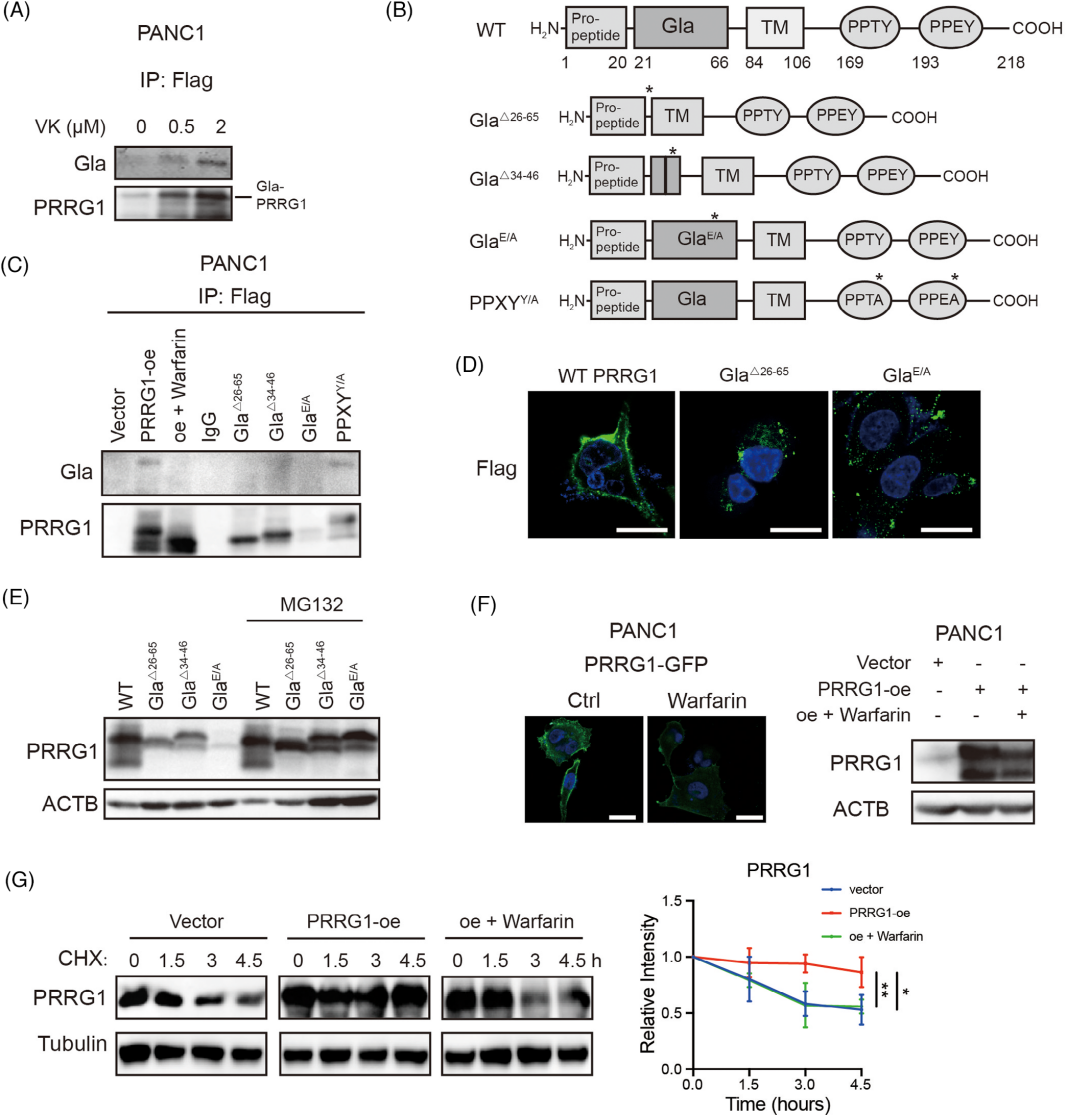
Gla modification is indispensable for the membrane localization and stability of PRRG1. (A) Identification of Gla modification using a specific antibody to Gla. PRRG1 is immunoprecipitated with the anti‐Flag antibody. Cells were pre‐cultured in serum‐free DMEM supplemented with ITS for 2 days, followed by the addition of vitamin K (0, .5, and 2 µM) for 3 h. (B) Schematic representation of wild‐type PRRG1 alongside truncated and mutant forms of PRRG1. (C) Coimmunoprecipitation of Flag and immunoblotting with antibodies against Gla modification or PRRG1in vector and PRRG1 over‐expressing PANC1 cells with or without warfarin, as well as PANC1 cells with truncated and mutant forms of PRRG1. (D) Representative immunofluorescence images show the specific localization of PRRG1 in PANC1 cells overexpressing wild‐type or PRRG1 variants. Scale bar: 25 µm. (E) Analysis of the level of PRRG1 with Gla‐domain truncated or mutated in the presence or absence of proteasome inhibitor MG132. (F) Representative immunofluorescence images of PRRG1 overexpressing PANC1 cells (GFP‐tagged), treated with or without warfarin (left). Western blot analysis of PRRG1 expression in vector and PRRG1 overexpressing (treated with or without warfarin) PANC1 cells (right). (G) Analysis of the protein stability of PRRG1 in PANC1 cells. Vector and PRRG1 over‐expressing (treated with or without warfarin) PANC1 cells were treated with 10 µg/mL CHX for the indicated times. The band densities of PRRG1 relative to Tubulin at the final CHX treatment time point were shown on the right. Mean ± SD, 1‐way ANOVA with Dunnett's post hoc test (*n* = 3 for each group). *p < .05, **p < .01. Oe, overexpression; GFP, green fluorescent protein; CHX, cycloheximide; TM, transmembrane; PPTY, proline‐proline‐threonine‐tyrosine; PPEY, proline‐proline‐glutamic acid‐tyrosine; PPTA, proline‐proline‐threonine‐alanine; PPEA, proline‐proline‐glutamic acid‐alanine; △26–65, the truncated form of PRRG1 missing the entire Gla domain from amino acid 26 to 65, and △34–46, missing the core element of the Gla domain from amino acid positions 34 to 46; E/A, Gla domain mutant form of PRRG1 that all glutamate encoding codons in Gla were replaced to those encoding alanine (A); Y/A, PPXY mutant form of PRRG1 where nucleotides encoding Y were replaced with those encoding A. Images in (A) and (C)–(F) are representative of two independent experiments.

The function and activities of vitamin K‐dependent proteins rely on proper γ‐carboxylation of the Gla domain.^15^ Two adjacent Gla residues of the core element of “EXXXE” can bind to one Ca^2+^ and enhance hydrophobicity for their interaction with phospholipids in plasma membranes. To characterize Gla modification of PRRG1, PRRG1‐Flag fusion constructs were generated and overexpression cells were established using the lentivirus expressing system. PANC1 cells overexpressing PRRG1‐Flag fusion protein were cultured in a serum‐free medium containing the insulin‐transferrin‐selenium (ITS) solution for 48 h. After that, the cells were treated with varying concentrations of vitamin K for 2 h. Subsequently, cells were lysed for immunoprecipitation using an anti‐Flag antibody. Immunoblotting was then performed with a specific antibody recognizing the modified Gla domain. As a result, it was ascertained that Gla modification of PRRG1 was indeed induced by vitamin K in a dose‐dependent manner (Figure 3A).

To investigate the critical role of the Gla domain in PRRG1, we generated truncated variants of PRRG1 lacking either the entire Gla domain (Gla^△26–65^) or key components of the Gla domain (Gla^△34–46^). Additionally, mutant forms of PRRG1 with altered Gla domain (Gla^E/A^) and PPXY motif (PPXY^Y/A^) were constructed, all fusion proteins were tagged with Flag, and their expression was achieved using the lentivirus system (Figure 3B). Gla modification was not detectable in these forms of PRRG1 with Gla domain deletion or mutation, while it remained in the wild‐type PRRG1 and PPXY^Y/A^ mutant form of PRRG1 (Figure 3C). Warfarin, an inhibitor of vitamin K epoxide reductase VKOR that blocks Gla modification, had been used for years in clinical practice for preventing thrombosis.^23^ We found that a low dose of warfarin that is reported to be insufficient to affect coagulation (2 µM),^24^ can effectively abolish Gla modification of PRRG1 in PANC1 cells (Figure 3C). The wild‐type PRRG1 exhibited predominant localization on the plasma membrane, whereas the Gla‐mutant or truncated PRRG1 was barely detectable on the PDAC cell surface (Figure 3D). In contrast to the wild‐type form, the Gla variants showed a propensity for rapid degradation, with their protein levels being strikingly increased after MG132 treatment (Figure 3E).

Furthermore, a plasmid encoding PRRG1 fused with a green fluorescent protein (GFP) was constructed, and an overexpression cell line was established in PANC1 cells. Fluorescence imaging of GFP demonstrated that treatment with warfarin markedly decreased the plasma membrane localization of PRRG1 and resulted in a reduction in its protein levels (Figure 3F). Additionally, the cycloheximide (CHX) assay illustrated that warfarin‐induced disruption of Gla modification diminished the stability of PRRG1 (Figure 3G). These findings suggest that Gla modification is essential for the membrane integration and protein stability of PRRG1.

### 2.4 PRRG1 overexpression promotes PDAC malignancy, which is reversed by low‐dose warfarin

**FIGURE 4:**
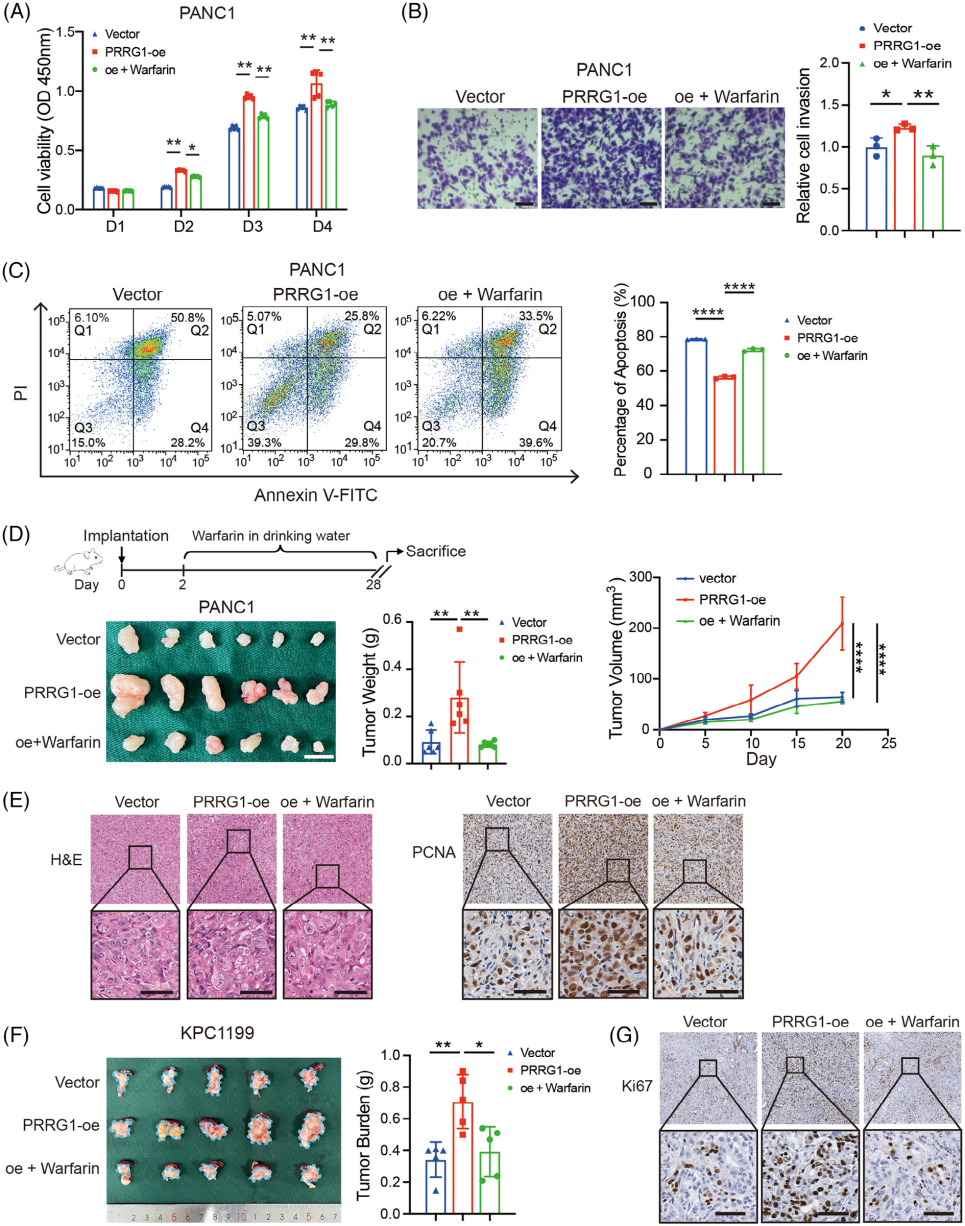
PRRG1 overexpression promotes PDAC progression which is eliminated by blocking Gla modification with low‐dose warfarin. (A) In vitro proliferation of vector and PRRG1‐overexpression PANC1 cells, treated with or without warfarin. Mean ± SD, two‐way ANOVA with Bonferroni's post hoc test (*n* = 3 for each group). (B) Cell migration in vector and PRRG1 overexpression PANC1 cells with or without warfarin. The relative cell number per field was analyzed using one‐way ANOVA with Dunnett's post hoc test (*n* = 3 for each group). Scale bar: 100 µm. (C) Analysis of apoptosis induced by serum‐free starvation in vector and PRRG1 overexpression (treated with or without warfarin) PANC1 cells. The percentages of annexin V‐positive apoptotic cells were analyzed using 1‐way ANOVA with Dunnett's post hoc test (n = 3 for each group). (D) Analysis of the effect of warfarin on in vivo tumour growth. After 48 h of inoculation, mice with PRRG1 overexpression cells were randomly divided into two groups and treated with or without warfarin (2 µmol/L) in normal drinking water. Photos of xenograft tumours (left); statistical analysis of tumour weight (middle), and growth curve of tumours over 20 days (right). Mean ± SD, one‐way ANOVA with Dunnett's post hoc test (*n* = 6 for each group). (E) Representative images of hematoxylin and eosin (H&E) and immunohistochemical staining of proliferating cell nuclear antigen (PCNA) in subcutaneous tumours derived from vector control and PRRG1‐overexpression PANC1 cells, with or without warfarin treatment. Scale bar: 50 µm. (F) Orthotopic tumour growth in mice inoculated with vector and PRRG1‐overexpression KPC1199^luc^ cells. Mice were similarly treated as described in (D). Representative images show orthotopic tumours circled with blue dots (left). Statistical analysis of the tumour burden is shown on the right (*n* = 5 for each group). (G) Representative images of immunohistochemical staining of ki67 in pancreas tissues bearing orthotopic tumours. Scale bar: 50 µm. The statistical significance of differences among groups was calculated by one‐way ANOVA with Dunnett's post hoc test or two‐way ANOVA with Bonferroni's post hoc tests. *p < .05, **p < .01.

To further validate that PRRG1 contributes to PDAC progression, we conducted both in vitro and in vivo experiments using cells that overexpress PRRG1. We also utilized a low dose of warfarin that disrupts Gla modification in the functional assays. PRRG1 overexpression led to increased capabilities of proliferation, anti‐apoptosis and migration, while warfarin evidently reversed all these effects in PANC1 cells (Figure 4A–C; Figure S3A,B). Further, we established a subcutaneous tumour model by inoculating the control and PRRG1‐overexpression PANC1 cells to the NOD‐SCID mice. Treatment with warfarin or vehicle control in drinking water started 2 days after inoculation and proceeded before harvesting. The result showed that PRRG1 overexpression significantly enhanced the in vivo tumour growth of PANC1 cells, which was clearly reversed by low‐dose warfarin (Figure 4D). Tumour growth was further confirmed by histological assessments with H&E staining and PCNA immunohistochemical staining (Figure 4E).

We also established a lentivirus‐mediated Prrg1 overexpression cell line using a mouse PDAC cell line expressing luciferase, named KPC1199^luc^. Consistently, Prrg1 overexpression also significantly promoted the proliferation of KPC1199^luc^ in vitro (Figure S3A,B). Furthermore, the cells were implanted into the pancreas of C57BL6 mice to generate a syngeneic orthotopic transplantation tumour model. Prrg1 overexpression significantly promoted the orthotopic tumour growth of KPC1199^luc^, as indicated by the increased bioluminescent signals and the pancreas burden (Figure 4F; Figure S3C). The increased tumour burden induced by Prrg1 overexpression was significantly attenuated after low‐dose warfarin treatment (Figure 4F; Figure S3C). Orthotopic tumour growth was further confirmed by histological assessments with H&E staining and Ki67 immunohistochemical staining (Figure 4G; Figure S3D). In conclusion, the results suggest that PRRG1 is a critical regulator of PDAC malignant progression which can be impeded by treatment of low‐dose warfarin.

### 2.5 PRRG1 interacts with NEDD4 through the PPXY motif and promotes NEDD4 self‐ubiquitination

**FIGURE 5:**
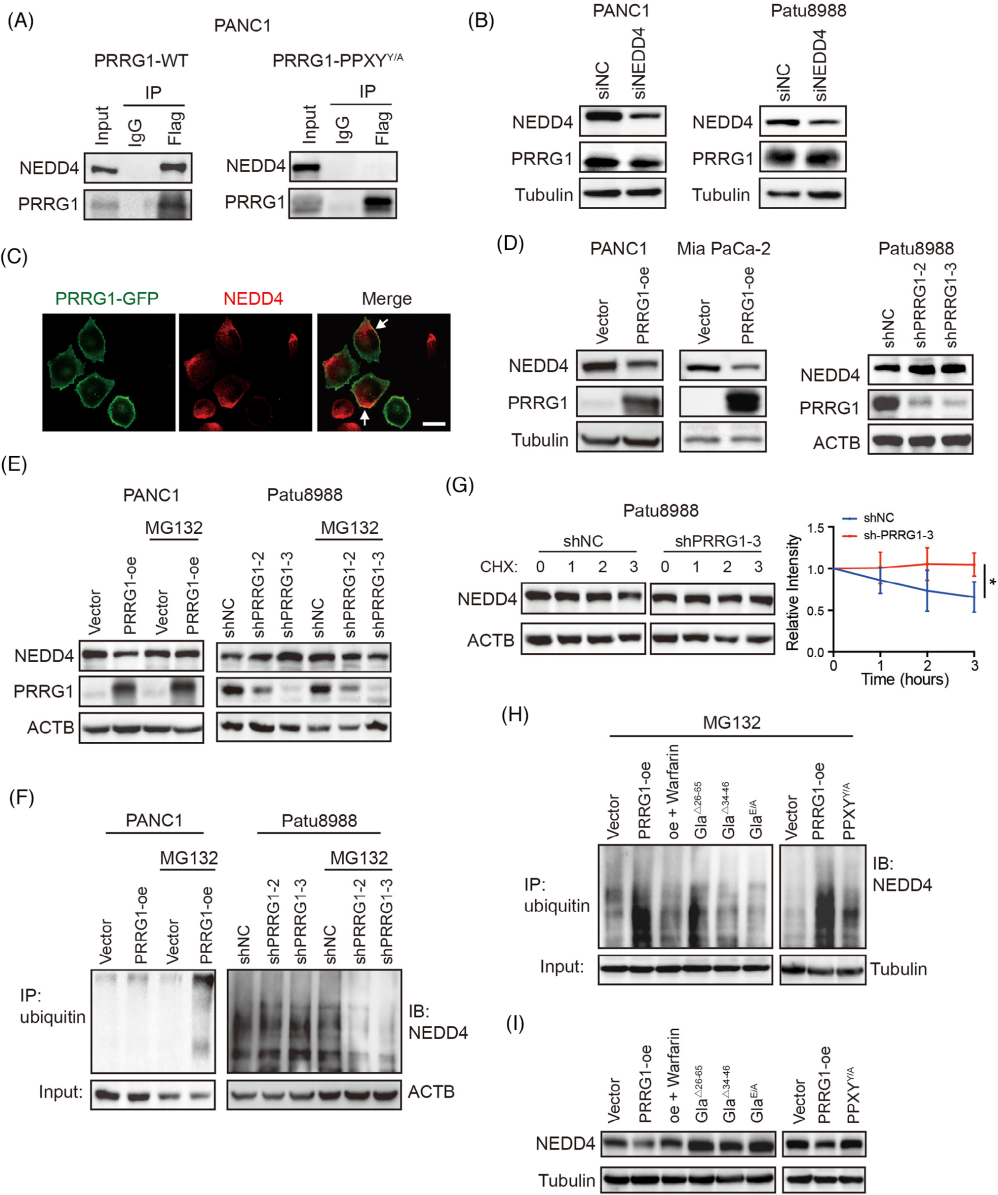
PRRG1 interacts with NEDD4 through the PY motif and promotes its self‐ubiquitination. (A) Coimmunoprecipitation of PRRG1 and NEDD4 in PANC1 cells expressing wildtype PRRG1 (left) or PPXY^Y/A^ mutant form of PRRG1 (right). (B) Western blot analysis of NEDD4 and PRRG1 expression in PANC1 cells (left) and Patu8988 cells (right), transfected with scrambled control or siRNA targeting NEDD4. (C) Representative co‐immunofluorescence images of PRRG1‐GFP and NEDD4 in PANC1 cells. White arrows indicate colocalized foci. Scale bar: 25 µm. (D) Western blot analysis of NEDD4 and PRRG1 expression in PRRG1‐overexpressed PANC1 cells (left) and Mia PaCa‐2 cells (middle), as well as PRRG1‐knockdown Patu8988 cells (right). (E) Western blot analysis of NEDD4 expression in vector and PRRG1‐overexpression PANC1 cells (left) and PRRG1‐knockdown Patu8988 cells (right), with or without MG132 treatment. (F) Immunoprecipitation of ubiquitin and immunoblotting with an antibody against NEDD4 in vector and PRRG1‐overexpression PANC1 cells (left), and in control and PRRG1‐knockdown Patu8988 cells (right), with or without MG123 treatment. (G) Analysis of the protein stability of NEDD4 in PRRG1 silencing and control Patu8988 cells. The band densities of NEDD4 relative to ACTB at the final CHX treatment time point were analyzed and shown on the right. Mean ± SD, the difference between groups were analyzed using an unpaired *t*‐test (*n* = 3 for each group). *p < .05. (H) Detection of NEDD4 ubiquitination in vector and PRRG1 overexpressing PANC1 cells with or without warfarin, as well as PANC1 cells expressing truncated or mutant forms of PRRG1 (with MG132 treatment). (I) Western blot analysis of NEDD4 expression in vector and PRRG1 overexpressing PANC1 cells with or without warfarin, as well as PANC1 cells expressing truncated or mutant forms of PRRG1. Images in (A)–(F), (H), and (I) are representative of two independent experiments.

To identify the PRRG1 binding partners in PDAC, we immunoprecipitated cell lysates of PRRG1‐Flag expressing PANC1 cells using an anti‐Flag antibody, then conducted a mass spectrometry analysis to determine the identities of the prey proteins. Some recognized interactors were identified including GGCX that mediates Gla modification. Several E3 ubiquitin ligases containing the WW domain that recognize PPXY motifs were also captured. As a plasma membrane‐anchoring E3 ligase that contains WW domains predictably recognizing PPXY motifs, NEDD4 was reported to have tumour suppressive roles through targeting Ras proteins or degradation.^9^ It also targets other membranous substrates for ubiquitination and degradation, including the epidermal growth factor receptors and vascular endothelial growth factor receptor 2.^25^, ^26^, ^27^, ^28^ Therefore, the interaction between PRRG1 and NEDD4 in PANC1 cells was confirmed through co‐immunoprecipitation and Western blotting assays (Figure 5A). To investigate whether the interaction between PRRG1 and NEDD4 depends on the intracellular PPXY motif, the PRRG1 mutant form PPXY^Y/A^ was coimmunoprecipitated with the anti‐Flag antibody. The findings revealed that the PPXY^Y/A^ mutant PRRG1 failed to establish complexes with NEDD4 (Figure 5A). Unexpectedly, the ubiquitination and the protein level of PRRG1 were not apparently altered by NEDD4 knockdown (Figure 5B; Figure S4), implying that binding to NEDD4 is not obligatory for PRRG1 degradation.

Both PRRG1 and NEDD4 were primarily localized on the plasma membrane, with their co‐localization confirmed through immunofluorescence analysis (Figure 5C; Figure S5). Intriguingly, the protein level of NEDD4 was clearly reduced by PRRG1 overexpression in both PANC1 and Mia PaCa‐2 cells, while it was obviously elevated in PRRG1‐knockdown Patu8988 cells (Figure 5D), indicating a clear negative regulation of NEDD4 by PRRG1. NEDD4/NEDD4L family protein was reported to undergo self‐ubiquitination and subsequent degradation upon binding to its targets.^29^ To investigate whether PRRG1 regulates NEDD4 through the ubiquitin‐proteasome system, the 26S proteasome inhibitor, MG132, was utilized concurrently. The diminished expression of NEDD4 resulting from PRRG1 overexpression was restored upon inhibition of ubiquitin proteolytic activity with MG132 (Figure 5E). Likewise, treatment with MG132 abolished the elevation of NEDD4 induced by PRRG1 knockdown (Figure 5E). Moreover, the ubiquitination of NEDD4 was clearly reduced in PRRG1‐knockdown cells compared with control cells, while it was increased by PRRG1 overexpression (Figure 5F). Consistently, the CHX experiment showed that NEDD4 stability was increased in PRRG1‐knockdown cells (Figure 5G). These results indicate that PRRG1 interacts with NEDD4 through the PPXY motif and promotes NEDD4 self‐ubiquitination and instability in PDAC cells.

To investigate the necessity of Gla modification for PRRG1 in regulating NEDD4 self‐ubiquitination, Gla‐mutant or deleted constructs were employed alongside the wild‐type PRRG1. The findings revealed that, unlike wild‐type PRRG1, PRRG1 variants lacking Gla modification did not enhance NEDD4 ubiquitination (Figure 5H) or reduce the protein level of NEDD4 (Figure 5I). Similar results were observed in low‐dose warfarin‐treated cells (Figure 5H,I). In conjunction with the results depicted in Figure 3, it is evident that Gla modification plays a crucial role in the membrane integration of PRRG1 to regulate the self‐ubiquitination of membrane‐bound NEDD4, which can be reversed by warfarin.

The NEDD4 family, comprising NEDD4 and NEDD4‐2, contains 3 to 4 WW domains that bind to the PY motifs (L/PPXY) of substrate proteins. It also includes a C‐terminal HECT domain, which is responsible for its ubiquitin ligase activity. Previous evidence has shown that the WW domains weakly bind to their own LPXY motif located within the HECT domain of NEDD4‐2, causing a “locked” stable state where the HECT domain remains inactive. This inhibitory WW‐HECT interaction is disrupted by higher‐affinity binding to proteins containing PPXY motifs, activating the HECT domain and initiating self‐ubiquitination.^29^ Aligning protein sequences revealed high similarity in domains and structure between NEDD4 and NEDD4‐2, with the HECT domain of NEDD4 containing the active‐site cysteine residue and conserved PY (LPXY) motif identical to its homolog NEDD4‐2.^30^, ^31^ Since PRRG1 possesses two PPXY domains (Figure 3B), it is highly probable to bind to the WW domains within NEDD4 through these motifs, triggering the “unlocking” of the ubiquitin ligase HECT domain and activating the self‐ubiquitination process, leading to the instability of NEDD4. To investigate whether the regulation of NEDD4 expression and ubiquitination relies on the PPXY motifs of PRRG1, PDAC cells overexpressing the PPXY^Y/A^ mutant form of PRRG1 were employed in the experiments. The findings showed that, in contrast to the wild‐type PRRG1 overexpression, the overexpression of PRRG1‐PPXY^Y/A^ did not enhance the ubiquitination of NEDD4 (Figure 5H), nor did it decrease the protein level of NEDD4 (Figure 5I). The above results demonstrate that PRRG1 promotes NEDD4 self‐ubiquitination and instability in a manner dependent on the C‐terminal PPXY motif.

### 2.6 PRRG1 knockdown reduces the signalling of oncoprotein KRAS and tyrosine kinase receptor EGFR

**FIGURE 6:**
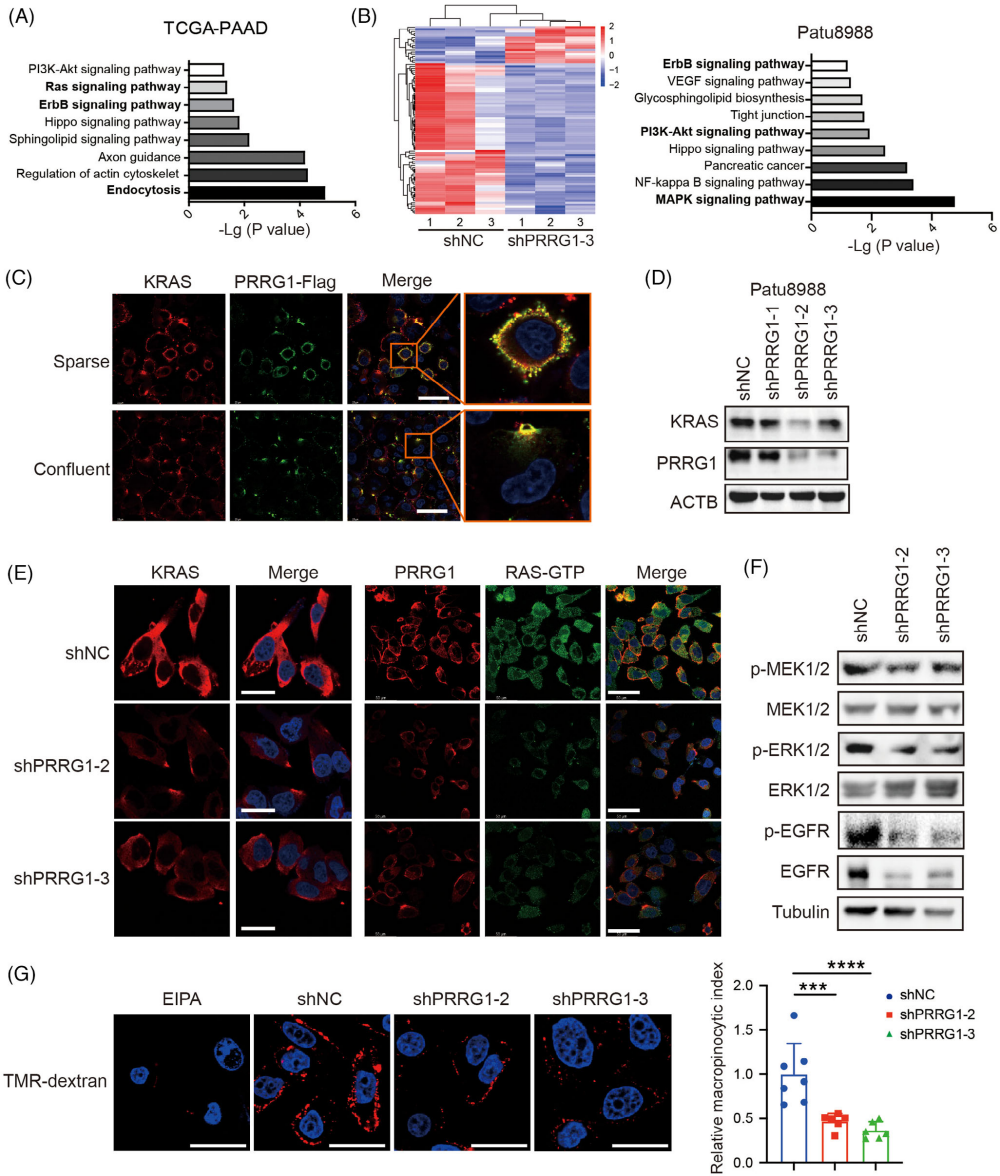
PRRG1 knockdown reduces the protein level of KRAS and EGFR, accompanied by decreased downstream signalling. (A) Gene ontology analyses of co‐expressed genes of PRRG1 in TCGA PAAD database. (B) Heatmap and KEGG analyses of differentially expressed genes of control and PRRG1 knockdown Patu8988 cells, as revealed by the transcriptome RNA‐sequencing. (C) Representative co‐immunofluorescence images of KRAS and Flag fused to PRRG1 and its regional magnification in sparse and confluent Patu8988 cells. Scale bar: 50 µm. (D) Western blot analysis of PRRG1 and KRAS expression in control and PRRG1 knockdown Patu8988 cells. (E) Immunofluorescence staining of KRAS (left) and co‐immunofluorescence staining of PRRG1 and active RAS‐GTP (right) in control and PRRG1 knockdown Patu8988 cells. Scale bar: 25 µm (left) and 50 µm (right). (F) The level of phospho‐MEK1/2, phospho‐ERK1/2, phospho‐EGFR (Tyr1068), and total MEK1/2, ERK1/2, and EGFR in control and PRRG1‐knockdown Patu8988 cells. (G) Representative immunofluorescence images of dextran macropinosomes (left) and relative macropinocytic index (right) in control and PRRG1‐knockdown Patu8988 cells. EIPA was used as a negative control of micropinocytosis. Scale bar: 25 µm. The statistical significance of differences among groups was calculated by one‐way ANOVA with Dunnett's post hoc test. ***p < .001; ****p < .0001. Images in (C)–(G) are representative of two independent experiments.

PRRG1 co‐expressed genes in the TCGA PAAD database were significantly enriched in endocytosis, Sphingolipid signalling pathway, Ras signalling, PI3K‐AKT and ErbB signalling (Figure 6A). GSEA analysis also exhibited a close correlation of PRRG1 with Ras signalling (Figure S6). Transcriptome RNA‐sequencing of control and PRRG1‐knockdown Patu8988 cells revealed a range of differentially expressed genes enriched in the MAPK signalling, Glycosphingolipid biosynthesis and PI3K‐AKT signalling (Figure 6B). In PDAC, mutant KRAS is sustained with a GTP‐binding active form, which acts as a driving factor to activate the tumour‐promoting signalling pathways like PI3K‐AKT, MAPK/ERK, and PLC‐NF‐kB pathways.^6^ The oncogenic KRAS signalling also acts in a positive feedforward loop with glycosphingolipids (GSLs).^32^ To address whether PRRG1 has relevance with KRAS signalling, we conducted the co‐immunofluorescence staining experiment and the result showed that PRRG1 was dominantly localized at the plasma membrane with considerable colocalization with KRAS both in sparse cells and confluent cells (Figure 6C). In confluent cells, concentrated colocalization of PRRG1 and KRAS was found in “cup”‐like microcompartments of the plasma membrane. Oncogenic mutant KRAS is anchored to the inner cell membrane and forms protein–lipid nanoclusters, which activate downstream effectors and induce macropinocytosis under nutrient‐deficient circumstances. We discovered that PRRG1 knockdown significantly reduced the expression level of KRAS (Figure 6D) as well as its aggregation in the plasma membrane of Patu8988 cells (Figure 6E). Immunofluorescence staining with an antibody specifically targeting the GTP binding form of Ras showed that active Ras‐GTP was also evidently attenuated by PRRG1 knockdown (Figure 6E). Coherently, the downstream phospho‐MEK1/2 (Ser217/221) and phospho‐ERK1/2 (Thr202/Tyr204) were clearly inhibited in PRRG1‐knockdown cells (Figure 6F). Mutant active KRAS promotes macropinocytosis, a highly conserved type of endocytosis, facilitating the non‐selective uptake of extracellular fluid to support PDAC development.^7^ In line with the regulation of PRRG1 towards KRAS, the capability of macropinocytosis was evidently suppressed by PRRG1 knockdown in serum‐free conditions, as determined by quantification of the internalized TMR‐dextran (Figure 6G).

It has been demonstrated that the tyrosine kinase receptor EGFR/ErbB‐1 formed a feed‐forward loop with oncogenic KRAS and was required for KRAS‐induced pancreatic tumorigenesis.^33^, ^34^ Intriguingly, we found that PRRG1 knockdown also suppressed the protein level and the active form of EGFR in PDAC cells, as indicated by Western blotting and immunofluorescence detection (Figure 6F; Figure S7). Collectively, these results indicate that PRRG1 knockdown decreases the protein level and signalling of both KRAS and EGFR in PDAC cells.

### 2.7 PRRG1 knockdown reduces the protein stabilities of KRAS and EGFR via NEDD4

**FIGURE 7:**
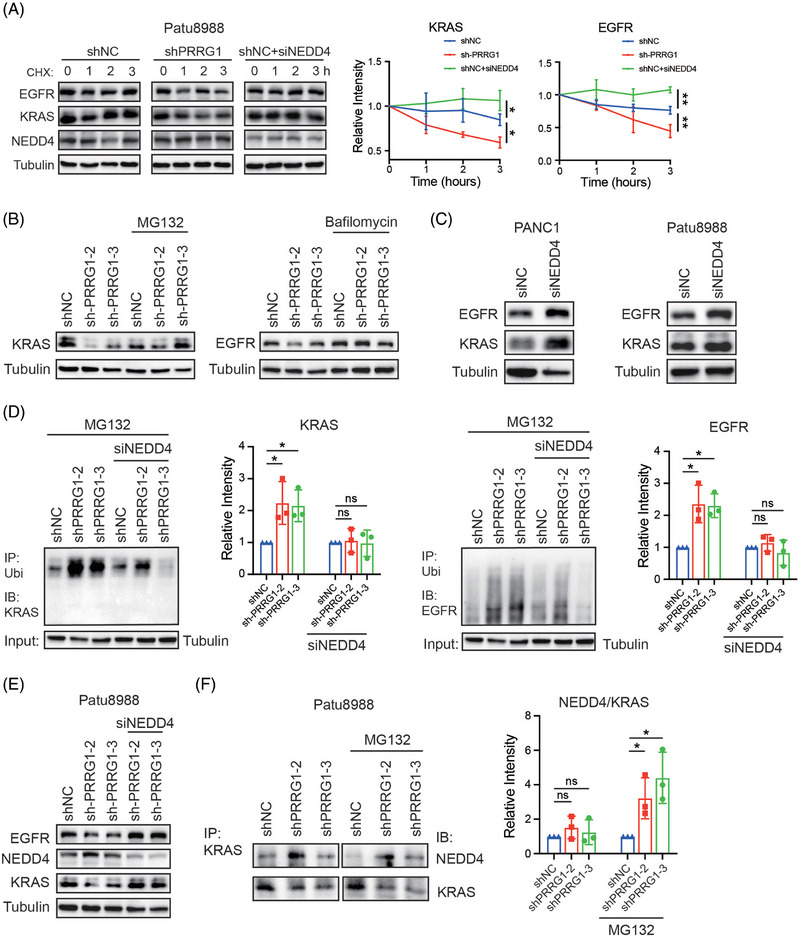
PRRG1 knockdown decreases the protein stability of KRAS and EGFR through elevating NEDD4. (A) Analysis of the protein stabilities of KRAS and EGFR in Patu8988 cells (left). Control and shPRRG1 Patu8988 cells (with or without siRNA‐mediated NEDD4 knockdown) were treated with CHX for the indicated times. The band densities of KRAS and EGFR relative to Tubulin at the final CHX treatment time point were shown on the right. Mean ± SD, one‐way ANOVA with Dunnett's post hoc test (*n* = 3 for each group). *p < .05. **p < .01. (B) Western blot analysis of KRAS expression in control and PRRG1‐knockdown Patu8988 cells with or without MG123 treatment (left), and EGFR expression in cells with or without Bafilomycin treatment (right). (C) Western blot analysis of the protein level of EGFR and KRAS in PANC1 cells (left) and Patu8988 cells (right) after NEDD4 knockdown. (D) Ubiquitination of EGFR (left) and KRAS (right) in shPRRG1‐Patu8988 cells with or without siRNA‐mediated NEDD4‐knockdown. Cells were pre‐treated with MG123 3 h before harvesting. The band densities of ubiquitinated EGFR and KRAS relative to Tubulin are shown beside. Mean ± SD, one‐way ANOVA with Dunnett's post hoc test (*n* = 3 for each group). *p < .05. (E) The protein levels of KRAS and EGFR were reduced in PRRG1‐knockdown Patu8988 cells, which were reversed by NEDD4 knockdown. (F) Coimmunoprecipitation of KRAS and NEDD4 in control and PRRG1‐knockdown Patu8988 cells treated with or without MG123. The band densities of coimmunoprecipitated NEDD4 relative to KRAS were analyzed and shown on the right. Mean ± SD, unpaired *t*‐test (*n* = 3 for each group). *p < .05. Images in (B), (C), and (E) are representative of two independent experiments.

NEDD4 binds to membranes and phospholipids in a Ca^2+^‐dependent fashion through its C2 domain. This interaction is critical for regulating the ubiquitin‐mediated endocytosis of several membrane proteins, including EGFR.^26^, ^35^, ^36^ NEDD4 has also been evidenced to mediate KRAS degradation through polyubiquitination on lysine 5. Considering the inhibitory regulation of PRRG1 toward NEDD4 described above, PRRG1 might contribute to stabilizing KRAS and EGFR through downregulating NEDD4. Indeed, the stabilities of KRAS and EGFR were clearly decreased in PRRG1‐knockdown cells as measured by the CHX assay, while they were increased by NEDD4 knockdown (Figure 7A). Blockade of proteasome‐mediated degradation with MG132 or lysosomal degradation with bafilomycin respectively eliminated the reduction of KRAS or EGFR by PRRG1 knockdown (Figure 7B). In contrast to the minimal modulation of PRRG1 (Figure 5B), NEDD4 knockdown clearly elevated the expression of KRAS and EGFR in PDAC cells (Figure 7C). Furthermore, the ubiquitination of KRAS and EGFR were increased in PRRG1‐knockdown cells, which were reversed by additional knockdown of NEDD4 (Figure 7D). Consistently, the reduction of the protein level of KRAS and EGFR in PRRG1‐knockdown cells was also rescued by NEDD4 knockdown (Figure 7E). In addition, enhanced interaction of NEDD4 with KRAS was detected in PRRG1‐knockdown cells, as determined by the semi‐quantification of the NEDD4/KRAS complex using the co‐IP/WB analysis (Figure 7F). These results indicate that PRRG1 exerts a protective role towards KRAS and EGFR through negatively regulating NEDD4 in PDAC cells.

### 2.8 Low‐dose warfarin reduced the protein levels of KRAS and EGFR that were elevated in PRRG1‐overexpressing cells

**FIGURE 8:**
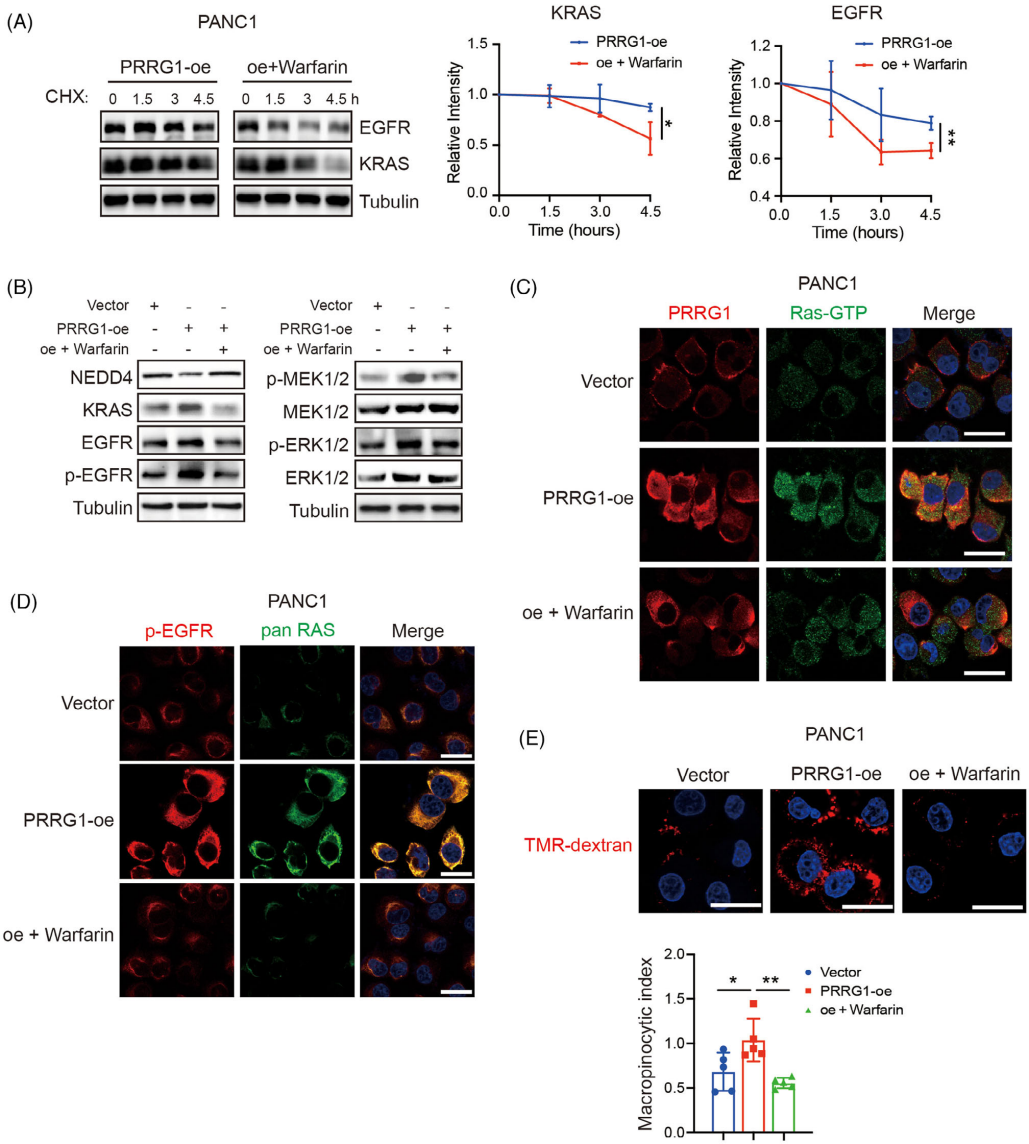
Low‐dose warfarin reduces the protein level and downstream signalling of KRAS and EGFR elevated by PRRG1 overexpression. (A) Protein stabilities of KRAS and EGFR in PRRG1‐overexpression cells treated with or without low‐dose warfarin. Mean ± SD, the relative band densities of KRAS and EGFR at the final CHX treatment time point were analyzed using an unpaired *t*‐test (*n* = 3 for each group). *p< .05; **p < .01. (B) The protein level of NEDD4, KRAS, EGFR and phospho‐EGFR (left panel), and the level of phospho‐MEK1/2, phospho‐ERK1/2, total MEK1/2, and ERK1/2 (right) in vector and PRRG1‐overexpression cells with or without warfarin treatment (2 µM). (C) Representative co‐immunofluorescence images of PRRG1 and Ras‐GTP in vector and PRRG1‐overexpression PANC1 cells (treated with or without treatment of 2 µM warfarin). Scale bar: 25 µm. (D) Representative co‐immunofluorescence images of p‐EGFR and pan‐Ras in vector and PRRG1‐overexpression PANC1 cells with or without warfarin. Scale bar: 25 µm. (E) Representative immunofluorescence images of the dextran macropinosomes (left) and statistical analysis of the relative macropinocytic index (right) in vector and PRRG1‐overexpression PANC1 cells (treated with or without warfarin). Scale bar: 25 µm. Mean ± SD, one‐way ANOVA with Dunnett's post hoc test (*n* = 5 for each group). *p < .05, **p < .01. Images in (B)–(D) are representative of two independent experiments.

We further investigated whether warfarin inhibits the upregulation of KRAS and EGFR in PRRG1‐overexpression cells. The CHX assay demonstrated that treatment of low‐dose warfarin, the antagonist of vitamin K and Gla modification, apparently decreased the protein stabilities of KRAS and EGFR in PANC1 cells (Figure 8A). As shown in Figure 8B, PRRG1 overexpression significantly increased the protein levels of KRAS and EGFR, accompanied by elevated levels of active phospho‐EGFR and the downstream MEK‐ERK signalling. All these signals were remarkably reversed by treatment of low‐dose warfarin, as detected by Western blotting (Figure 8B). Additionally, immunofluorescence staining showed that the active Ras‐GTP and phospho‐EGFR were reinforced in PRRG1‐overexpressing cells compared with vector‐control cells, which were clearly reversed by low‐dose warfarin (Figure 8C,D). Warfarin also reversed the increased macropinocytosis in PRRG1‐overexpression cells, as detected by quantitative of the internalized TMR‐dextran in serum‐free conditions (Figure 8E).

Together, these results indicate that PRRG1 protects the membranous signalling of KRAS and EGFR through counteracting E3 ligase NEDD4 in PDAC cells, which can be blocked by inhibitors targeting vitamin K‐dependent Gla modification.

## 3 DISCUSSION

Besides oncogenic gene mutations (KRAS, TP53, CDKN2A, etc.), aberrant overexpression of transmembrane proteins, including growth factor receptors, iron channels, and adhesion proteins, are crucial for the activation of oncogenic pathways and PDAC tumorigenesis.^37^ Data mining of PDAC databases demonstrated a prominent correlation of membrane molecules with PDAC prognosis. In this study, we unveil both the expression and the functional significance of PRRG1, a single‐pass transmembrane protein, in PDAC progression. The γ‐glutamic acid carboxylation of PRRG1 dependent on vitamin K was identified in PDAC cells, which was proved to be critical for PRRG1 membrane association and molecular function. Mechanically, PRRG1 exerted a protective role toward EGFR and oncogenic KRAS‐MEK‐ERK signalling via inhibiting NEDD4, a membrane‐associated E3 ligase that mediated the ubiquitination of KRAS and EGFR.^38^ Moreover, a low dose of warfarin below the anticoagulant dosage, effectively blocks γ‐carboxylation of the Gla domain of PRRG1, reverses its regulation on KRAS and EGFR, and PDAC in vivo tumour growth. Therefore, we identified PRRG1 as a key regulator of PDAC progression, highlighting the importance of two featured modules, the Gla domain and the PPXY motif, on its molecular function.

We observed that anchorage‐independent growth and in vivo, tumour growth were more dramatically inhibited by PRRG1 knockdown cells as compared with that in the in vitro proliferation assay. Unlike in vivo tumour growth, cells cultured in monolayers adhere to rigid substrates and the extracellular matrix. Apart from oncogenic signalling, cell‐matrix interaction‐mediated mechanical signalling pathways also play a role in regulating cell proliferation.^39^ Anchorage‐independent growth, is a signature of oncogene‐induced transformation and tumour progression. Transformed cells exhibit the ability to survive and proliferate without attachment, a key trait in cancer metastasis. Notably, transformed tumour cells with oncogenic KRAS mutations display a greater dependency on KRAS in anchorage‐independent cultures compared with monolayer cultures.^40^ Given the protective role of PRRG1 on oncogenic KRAS, it is conceivable that PDAC cells rely more heavily on PRRG1 for growth under anchorage‐independent conditions. These findings also suggest the potential significance of PRRG1 in PDAC metastasis, an area warranting further exploration through in vivo metastasis models.

Unlike the Gla domain‐containing coagulant and anticoagulant factors, PRRGs were reported to be expressed in several extrahepatic tissues and no role in coagulation had yet been ascribed to them.^20^ In normal pancreas tissue, we found that PRRG1 was nearly undetectable except for mild expression in islet cells, while it was upregulated in PDAC cells with prognosis significance. PRRG1 expression was highly correlated with Ras and ERBB signalling in TCGA PAAD tissues, but the regulatory mechanism of PRRG1 expression in PDAC remained unexplored in this study. Whether it is regulated by or forms a positive feedback loop with these oncogenic signals deserves further investigation. Although PRRG1 colocalized and interacted with membrane‐anchoring NEDD4, the protein stability of PRRG1 was not increased after NEDD4 knockdown. Thus, NEDD4 was probably not the primary E3 ligase obligatory for PRRG1 degradation in PDAC cells.

The ubiquitin‐related ligases of the Nedd4/Rsp5p family comprise a Ca^2+^‐dependent lipid‐binding (C2) domain in the N‐terminal, multiple WW domains, and a functional HECT domain in the C‐terminal. NEDD4 played a crucial role in the ubiquitination process of membranous receptors (e.g., EGFR), transporters (e.g., the epithelial Na^+^ channel, ENac), and plasma membrane‐associated proteins (K‐Ras, H‐Ras), which were subsequently subjected to proteasome‐mediated degradation or endocytosis for lysosomal degradation.^35^ In PDAC development, oncogenic KRAS and EGFR signal constituted a positive feedback loop to drive malignant transformation, both of which were characterized as substrates of NEDD4.^33^, ^34^ By knocking down or overexpressing PRRG1, we repeatedly found an evident negative regulation of NEDD4 by PRRG1 in different PDAC cells, accompanied with positive regulation of KRAS and EGFR signalling. Further evidence indicated that PRRG1 exerted a protective role on the membrane signal of KRAS and EGFR through downregulating E3 ligase NEDD4. Nevertheless, PRRG1 might also interact with other extracellular or cytoplasmic proteins, or have other biological roles in different contexts, which is to be revealed in future.

PRRG1 expression was also closely correlated to the sphingolipid pathway in PDAC (Figure 6A,B). Recently, a reciprocal interdependence of KRAS function and anaerobic glycolysis‐involved synthesis of defined GSL in PDAC cells was reported. The carcinogenic mutation of KRAS led to enhanced synthesis of GSLs, which were inserted into the outer leaflet of the cell membrane. Reciprocally, GSLs mediated a trans‐bilayer lipid coupling to maintain the phosphatidylserine content in the inner leaflet and potentiated KRAS clustering and oncogenicity.^32^ Sphingolipids are composed of a hydrophobic ceramide skeleton and hydrophilic glycosyl groups. According to a literature report, the presence of sphingolipids can multiply the affinity between the Gla domain‐containing anticoagulation factor protein C and phospholipid vesicles.^41^ Presumably, PRRG1 might regulate the formation of the trans‐bilayer GSL‐KRAS nanoclusters through its Gla domain, yet the underlying mechanism is to be further explored.

As an ancient post‐translational modification, Gla residues play a crucial role in regulating the vitamin K‐dependent protein family.^42^ Their ability to interact with phospholipid membranes via Ca^2+^ might make them attractive candidates as signalling molecules for cellular communications. The bidentate Gla residues together with Ca^2+^ might be involved in the formation of molecular scaffolds for intracellular or extracellular matrices.^43^ GAS6 was the only well‐characterized Gla protein that regulated oncogenic signalling pathways through binding to and activating Axl receptor tyrosine kinase on tumour cells, which was overexpressed in several types of cancer.^44^ Our study characterized PRRG1 as another Gla protein that played crucial roles in oncogenic tumour growth in PDAC, with its function dependent on the γ‐carboxylation of the Gla domain. Cancers were usually featured with a hypercoagulable state and pancreatic cancers were associated with significant thrombophilia.^45^ Warfarin usage has been reported to be linked with improved overall survival in certain cancers, with growing evidence supporting its anti‐tumour effects via both blood plotting pathway‐dependent and independent mechanisms, the latter of which were evidenced in the inhibition of the Gas6‐AXL signalling.^18^, ^46^, ^47^ Our results demonstrated that usage of low‐dose warfarin effectively blocked KRAS and EGFR signalling, two vital regulators of PDAC carcinogenesis, by disrupting the Gla protein PRRG1. Therefore, targeting PRRG1 with warfarin or other reagents may constitute a promising therapeutic strategy for PDAC.

## 4 METHODS AND MATERIALS

### 4.1 Data mining using gene expression omnibus and TCGA

The expression dataset from the TCGA genomic project (https://portal.gdc.cancer.gov) is retrieved for investigating the mRNA expression of KRAS‐related genes in PDAC. To discover the upregulated genes in PDAC with poor prognosis, the expression profiles of GSE15471, GSE16515, and GSE28735 from GEO (https://www.ncbi.nlm.nih.gov/geo) were downloaded, as well as a gene list significantly linked to poor prognosis in PDAC from OncoLnc (http://www.oncolnc.org).

To better compare the expression level of PRRG1 in PDACs and normal counterparts, we retrieved the PRRG1 expression profiles of GSE15471 (with 39 paired PDAC tumours and matching normal pancreatic tissue samples), GSE16515 (with 36 tumours and 16 adjacent tissues, all derived from patients diagnosed with stage II or III pancreatic adenocarcinoma), GSE28735 (with 45 paired PDAC tumours and matching normal pancreatic tissue samples) from GEO, as well as from TCGA databases and GTEx (https://gtexportal.org). For analyzing the prognosis value of PRRG1, the clinical data were compiled from TCGA PDAC patients, correlating survival outcomes with PRRG1 mRNA levels.

Patients were grouped into two categories based on their expression levels. In short, the lowest 71 cases were classified as having “low” expression, while the highest 72 cases were designated as “high” expression. Also, we analyzed disease‐free survival using the TCGA PDAC dataset, splitting patients into nonoverlapping groups of 36 cases each, representing the top and bottom quartiles of expression levels, respectively.

### 4.2 Patients and tissue samples

A total of 150 PDAC patients who underwent radical surgery at Ren Ji Hospital, School of Medicine, Shanghai Jiao Tong University, Shanghai, China, between January 2017 and December 2018 were included in the study. Only patients with complete follow‐up records spanning more than 2 years were enrolled, while those who died due to perioperative complications were excluded. Tumours were staged according to the TNM classification from the American Joint Committee on Cancer (AJCC), 8th edition.^48^ The follow‐up data encompassed the patients’ demographic information, pathology reports, surgical details, and survival durations. Resected specimens from surgery were characterized by tumour size, tumour location, tumour invasion, lymph node status, and vascular invasion. As a result, 84 patients with complete data were included in the Renji cohort. All samples were obtained from patients who had provided informed consent prior to the operation in Renji Hospital. And protocols were authorized by the ethical review committee of the World Health Organization Collaborating Center for Research in Human Production (approved by the Shanghai Municipal Government).

### 4.3 Cell lines and cell culture

We purchased the human pancreatic cancer cell lines PANC1, Patu8988. and MIA PaCa‐2 from the Cell Bank of the Chinese Academy of Sciences. Additionally, the primary mouse tumour cell line KPC1199 was obtained from KPC (LSL‐KrasG12D/+; LSL‐Trp53R172H/+; Pdx‐1‐Cre, C57BL/6 background) pancreatic ductal adenocarcinoma mouse model which was generously provided by Prof. Jing Xue (Renji Hospital). We cultured all cells in Dulbecco's modified Eagle's medium (DMEM) added with 10% (v/v) fetal bovine serum. The cells were cultured with 1% antibiotics and incubated at 37°C in a humidified chamber containing 5% CO2.

### 4.4 Small interference RNA‐mediated knockdown of gene expression

siRNAs were transfected into PDAC cells to specifically knock down gene expression. The jetPRIME transfection reagent (Polyplus) was used following the manufacturer's guidelines. After 72 h, the cells were harvested, and Western blotting was performed to evaluate the efficiency of the knockdown. The effective siRNA sequences can be found in Table S3.

### 4.5 Lentivirus‐mediated overexpression and knockdown of PRRG1

Full‐length cDNAs encoding human PRRG1 or mutant forms of PRRG1 including Gla domain truncated (Gla^△26–65^ and Gla^△34–46^), Gla‐domain mutant (Gla^E/A^) and PPXY mutant (PPXY^Y/A^) were synthesized and cloned to the pSLenti‐CMV‐MCS‐3xFlag‐PGK‐puro‐WPRE plasmid. For stable knockdown of PRRG1, the PLKO.1 vector (Invitrogen) was used to construct the plasmid expressing short hairpin RNAs targeting PRRG1 (shPRRG1). The shRNA‐encoding sequences targeting PRRG1 are listed in Table S4. We generated the lentiviral particles by co‐transfection of PRRG1 or mock vectors in 293T cells with the three‐plasmid packaging system (pPACKH1‐REV, pPACKH1‐GAG, and pVSV‐G), with the help of Lipofectamine 2000 (Invitrogen, 11668‐019). After transfection, we collected viruses and then measured them at both 48 and 72 h. Cells were infected with recombinant lentivirus‐transducing units accompanied by Polybrene for 5 µg/mL, (Sigma‐Aldrich, H9268). After 48 h of infection, we cultured cells for 14 days with 5 µg/mL puromycin (Gibco, A1113802) to eradicate uninfected cells, then further in a medium containing 1 µg/mL puromycin.

### 4.6 Animal studies

For constructing the subcutaneous xenograft model, we subcutaneously injected 6 × 10^6^ Patu8988 cells in the flank of 6‐week‐old female M‐NSG immunodeficient mice. The cells were expressing stably shNC or sh‐PRRG1‐2 or sh‐PRRG1‐3. Tumours were measured with a calliper every week and the tumour volumes (*V*) were calculated with the formula: *V* = .52 × *L* (tumour length) × *W* ^2^ (tumour width). The mice were sacrificed to collect and dissect tumours for further measurements after growing for 4 weeks.

The mice were housed in isolated, ventilated cages at the Shanghai Jiaotong University Laboratory Animal Center. They were kept under a 12 h light/dark cycle, with temperatures between 22°C and 26°C. Water and sterile pellet food were provided ad libitum. This study followed the guidelines outlined in the Guide for the Care and Use of Laboratory Animals and applicable Chinese regulations. The protocol was approved by the Institutional Animal Care and Use Committee (IACUC) at Shanghai Jiaotong University under Animal Protocol number A2023083.

### 4.7 Histological analysis

Phosphate‐buffered formalin was used to fix tissues and then embedded in paraffin for sectioning. The slides underwent H&E staining or immunohistochemical staining using antibodies with cell proliferating marker Ki‐67 (GB111141, rabbit, Servicebio), PCNA (GB11010, rabbit, Servicebio), or TUNEL labelling of apoptotic cells. For immunohistochemical staining of PRRG1 in the PDAC tissue microarray, the antibody of PRRG1 (PA5‐67490, rabbit, Invitrogen) was used. As previously described, PRRG1 expression was scored according to the intensity and proportion of positive cells. The scoring system was as follows: 0 for 0–5% positivity, 1 for 6–35% positivity, 2 for 36–70% positivity, and 3 for more than 70% positivity.^49^, ^50^

### 4.8 Cell proliferation, apoptosis, and transwell‐based migration assays

For the cell proliferation assay, all experiments were conducted as previously outlined.^51^ In Brief, cells were plated in a 96‐well plate for later treatment with cell counting kit‐8 (Share‐bio). One hour after CCK8 treatment, the absorbance of cells at 450 nm was recorded with a BioTek Synergy HTX Multimode Reader (Agilent). Measurements were taken 24, 48, 72, and 96 h after cell plating.

For the apoptosis assay, cells were cultured in a six‐well plate and deprived of serum to promote apoptosis. After 48 h, cells were stained with propidium iodide and annexin V‐FITC (Share‐bio). We determined apoptosis in cells with flow cytometry using a BD FACSCalibur instrument (BD Biosciences).

For the transwell‐based migration assay, we cultured 2 × 10^4^ cells on the upper side of the chamber in the serum‐free DMEM medium. As a normal chemoattractant, the lower chamber was added with DMEM medium containing 10% FBS. After a 24 h incubation, cells that did not migrate were carefully removed from the upper surface of the membrane using a cotton swab. The cells that migrated to the lower side of the filter were then fixed with 4% paraformaldehyde for crystal violet staining. Finally, migration was evaluated under a microscope, and the number of migrated cells was quantified from three randomly selected fields in each of the three replicate experiments.

### 4.9 The soft agar colony formation assay

First, we applied a 1.2% agarose (Share‐bio) layer to the bottom of 6‐well plates in DMEM medium containing 10% FBS. Next, 10 000 cells were combined with .6% agarose and placed as the top layer. Two‐hundred microlitre medium was placed on the top agarose layer and replaced every 3 days. We cultured the plate at 37°C with 5% CO2 for 4 weeks, allowing the colonies to form. Afterwards, colonies were stained with 200 µL of MTT (0.5 mg/mL) for 30 min in an incubator. Colonies were quantified with three replicates.

### 4.10 Detection and quantification of macropinosome

Macropinosome visualization experiments were carried out as previously noted.^52^ Briefly, we incubated cells on an Ibidi 8‐well culture slide in starvation with serum‐free medium for 18 h. To visualize macropinosomes, a 70 kDa high‐molecular tetramethylrhodamine–dextran (TMR‐dextran, D1818, Invitrogen) was added to the serum‐free medium at 1 mg/mL for 40 min in the incubator. After that, we rinsed the cells with cold PBS five times (5 min for each rinse) and then fixed them with 4% paraformaldehyde. Cellular internalization of TMR‐dextran was captured with a confocal microscope (Leica). We randomly counted at least 200 cells for quantitative analysis. We analyzed the images using the ImageJ software (ImageJ). The macropinocytic index is calculated as the total fluorescent area of TMR‐dextran divided by the total cell area, multiplied by 100.

### 4.11 Transcriptome RNA‐sequence

Total RNA from control and PRRG1 knockdown Patu8988 cells (three biological replicates) was extracted. The mRNA was harvested by RNeasy Mini Kit (Qiagen). Libraries were constructed with a TIANSeq Fast RNA Library Prep Kit (TIANGEN). Illumina NovaSeq 6000 platform was used to sequence the library preparations. We use the DESeq2 R package to analyze differentially expressed genes in the transcriptome. Genes with fold change greater than 1.5 and *p*‐values below .05 were considered significantly different. We used the DAVID functional annotation tool to analyze KEGG pathway enrichment results. KEGG terms with adjusted *p*‐values below .05 were regarded as significantly enriched. The accession number for the RNA‐seq data is GSE273196.

### 4.12 Reagents and inhibitors

To determine protein stability, we treated cells with cycloheximide (C7698, Sigma‐Aldrich) at a concentration of 10 µg/mL for 0, 1, 2, and 3 h; or for 0, 1.5, 3, and 4.5 h prior to harvesting. Cell lysates were subjected to Western blotting. We assessed the relative protein levels by quantification of the band density of the specific protein in comparison with the internal control. To investigate whether the alteration of the protein level was attributed to the ubiquitin‐proteasome system, we added MG132 (HY‐13259, MedChemExpress) or vehicle control to the medium at a final concentration of 10 µM for 2 h. To detect whether the lysosomal protein degradation system was involved, Bafilomycin A1 (HY‐100558, MedChemExpress) was applied. To block vitamin K‐dependent Gla modification, warfarin (S4545, Selleck) was utilized. Ethylisopropylamiloride (EIPA, HY‐101840, MedChemExpress) was used as an inhibitor of micropinocytosis.

### 4.13 Co‐immunoprecipitation and mass spectrometry

To identify proteins that bind to PRRG1, we performed immunoprecipitation (IP) with the anti‐DDDDK tag (FLAG tag) antibody (66008‐4‐Ig, mouse, Proteintech) with Protein A/G Magnetic Beads (B23202, Selleck). Samples from PANC1 and Patu8988 cells were processed for SDS‐PAGE electrophoresis, followed by staining with Coomassie blue as well as mass spectrometry (MS) analysis using an Easy nLC1200 system (Thermo Scientific). For verifying the specific interacting proteins, we incubated cell lysates with magnetic beads conjugated with DDDDag antibody or IgG for 2 h at 25°C. After that, we washed the beads for 5 min with PBS/Tween‐20 three times before releasing the immunoprecipitation mixtures with 1*loading buffer. Finally, we performed Western blotting on the protein lysates to detect specific antibodies.

### 4.14 Protein ubiquitination

To assess the ubiquitination levels in specific proteins, we pretreated cells with MG132 for 2 h and harvested using lysis buffer (P70100, NCM Biotech) containing protease and phosphatase inhibitor cocktail (P002, NCM Biotech). We performed immunoprecipitation (IP) on the protein lysates using either an anti‐ubiquitin antibody (10201‐2‐AP, rabbit, Proteintech) or an anti‐DDDDK (FLAG tag) antibody (66008‐4‐Ig, mouse, Proteintech), followed by capture with protein A/G MAGNETIC BEAds (B23202, Selleck). After incubation, we analyzed the immunoprecipitated samples using SDS‐PAGE electrophoresis followed by western blotting. The samples immunoprecipitated with the anti‐ubiquitin antibody were probed using anti‐KRAS, anti‐EGFR, or anti‐PRRG1 antibodies. For the samples immunoprecipitated with the anti‐Flag antibody, an anti‐ubiquitin antibody (10201‐2‐AP, rabbit, Proteintech) was used for probing.

### 4.15 Immunofluorescence staining

For immunofluorescence staining, cells cultured on Ibidi 8‐well culture slides were fixed for 10 min with 4% paraformaldehyde. Next, cells were permeabilized with .1% Saponin (Abmole Bioscience, M9618) and then blocked with 1% BSA at 25°C. Primary antibodies against PRRG1 (PA5‐67490, rabbit, Invitrogen), Flag tag (66008‐4‐Ig, mouse, Proteintech), NEDD4 (21698‐1‐AP, rabbit, Proteintech), Ras‐GTP (26909, mouse, NewEast Bioscience), KRAS (12063‐1‐AP, rabbit, Proteintech), pan Ras (60309‐1‐Ig, mouse, Proteintech), and phospho‐EGFR Tyr1068 (3777s, rabbit, CST) were used for incubation at 4°C overnight. Subsequently, species‐matched secondary antibodies conjugated to Alexa Fluor‐488 or ‐594 were applied. The nucleus was stained with Hoechst and then sealed by an anti‐fade mounting medium (Beyotime, P0126). We captured immunofluorescence images using a confocal microscope (Leica).

### 4.16 Western blotting

Cells were lysed with lysis buffer (P70100, NCM Biotech) containing Phosphatase and Protease Inhibitor Cocktail (P002, NCM Biotech). We performed protein extraction and separation using SDS‐PAGE, followed by transfer onto a .22 µm pore‐size nitrocellulose membrane. The membrane was incubated with 5% non‐fat milk for 1 h to block nonspecific binding sites. Then, we performed the incubation of primary antibodies targeting PRRG1 (14103‐1‐AP, rabbit, Proteintech), Gla (3570, mouse, BioMedica Diagnostics), NEDD4 (21698‐1‐AP, rabbit, Proteintech), KRAS (12063‐1‐AP, rabbit, Proteintech), beta Actin (GB15001, mouse, Servicebio), MEK1/2 (11049‐1‐AP, rabbit, Proteintech), phospho‐MEK1/2 (Ser217/221) (9154T, rabbit, CST), ERK1/2 (11257‐1‐AP, rabbit, Proteintech), phospho‐ERK1/2 (Thr202/Tyr204) (80031‐1‐RR, rabbit, Proteintech), EGFR (sc‐373746, mouse, Santa Cruz), and phospho‐EGFR (Tyr1068) (3777s, rabbit, CST) at 4°C overnight, and then exposed to species‐specific secondary antibodies (7074P2, rabbit, and 7076P2, mouse, CST).

### 4.17 Statistical analysis

All statistical analyses were conducted with a minimum of three independent experiments, and the data are presented as mean ± SD. Related graphs were drawn in GraphPad Prism 10 software (GraphPad Software, Inc). To determine statistical significance across multiple experimental groups, we conducted one‐way analysis with Dunnett's post hoc test or two‐way analysis with Bonferroni's post hoc test. Clinical data were analyzed using SPSS 16.0 for Mac (IBM). Cumulative survival times were estimated through the Kaplan–Meier method, and comparisons were made using the log‐rank test. The relationship between PRRG1 expression and clinical features in PDAC patients was assessed via Fisher's exact test or the *χ*2 test. Statistical significance was set at a *p*‐value of less than .05.

## AUTHOR CONTRIBUTIONS

Zheng Wu, Qing Ye, Shan Zhang, Shu‐Yu Xiao, and Zong‐Hao Duan performed the experiments and statistical analysis. Zheng Wu and Xiao‐Qi Wang contributed to bioinformatics analysis. De‐Jun Liu provided clinical specimens and made clinical pathology evaluations. Shan Zhang, Li‐Peng Hu, Lei Zhu, Xue‐Li Zhang, and Lin‐Li Yao contributed to animal experiments. Shu‐Heng Jiang and Zhi‐Gang Zhang provided critical reviews. Xiao‐Mei Yang and Zheng Wu wrote the manuscript; Xiao‐Mei Yang, De‐Jun Liu, and Dong‐Xue Li conceived, designed, supervised, analyzed, and interpreted the study and provided critical reviews.

## CONFLICT OF INTEREST STATEMENT

The authors declare no conflict of interest.

## ETHICS STATEMENT

This study was conducted in accordance with the Guide for the Care and Use of Laboratory Animals and relevant Chinese laws and regulations. The protocol was approved by the Institutional Animal Care and Use Committee (IACUC) of Shanghai Jiao Tong University (animal protocol no. A2023083). All human samples were obtained from patients who provided informed consent prior to surgery at the Department of Biliary‐Pancreatic Surgery, Renji Hospital, School of Medicine, Shanghai Jiao Tong University. The protocols for human research were approved by the Ethical Review Committee of the World Health Organization Collaborating Center for Research in Human Reproduction, authorized by the Shanghai Municipal Government.

## Supporting information



Supporting Information

## Data Availability

Data supporting the findings of this study are available from the corresponding author upon reasonable request. The RNA‐sequencing data have been deposited in the GEO database under accession number GSE273196.
