# Supplementary material for: Vitamin K‐dependent gamma‐carboxyglutamic acid protein 1 promotes pancreatic ductal adenocarcinoma progression through stabilizing oncoprotein KRAS and tyrosine kinase receptor EGFR
Source: Clin Transl Med. 2025 Jan 22;15(1):e70191. doi: 10.1002/ctm2.70191 (PMC11753899; doi:10.1002/ctm2.70191)
Supplement: Supplementary file 1 — Supporting Information [file CTM2-15-e70191-s001.docx]

**Supplementary Figures and Tables**

**Supplementary Figures**


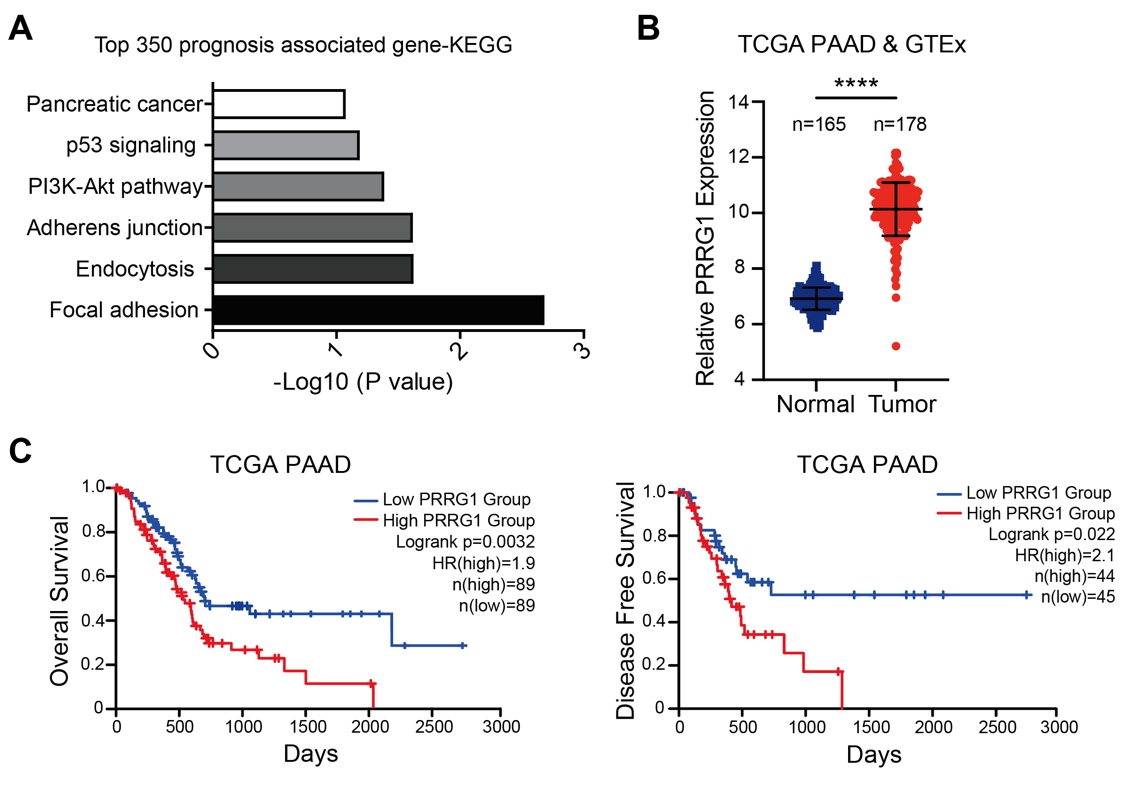


Supplementary Figure 1. (A) Kyoto Encyclopedia of Genes and Genomes (KEGG) pathway enrichment of the top 350 genes positively related to the prognosis of TCGA-PAAD patients. (B) PRRG1 mRNA level in PAAD cancer tissues vs normal tissues. Data was analyzed from TCGA PAAD dataset and GTEx databases. (C) Kaplan–Meier plot of the overall survival and disease-free survival of PDAC patients based on the expression of PRRG1, using TCGA PAAD databases. The statistical tests used were: gene expression analysis, unpaired t test; patient survival, log-rank test. ****P <0.0001.


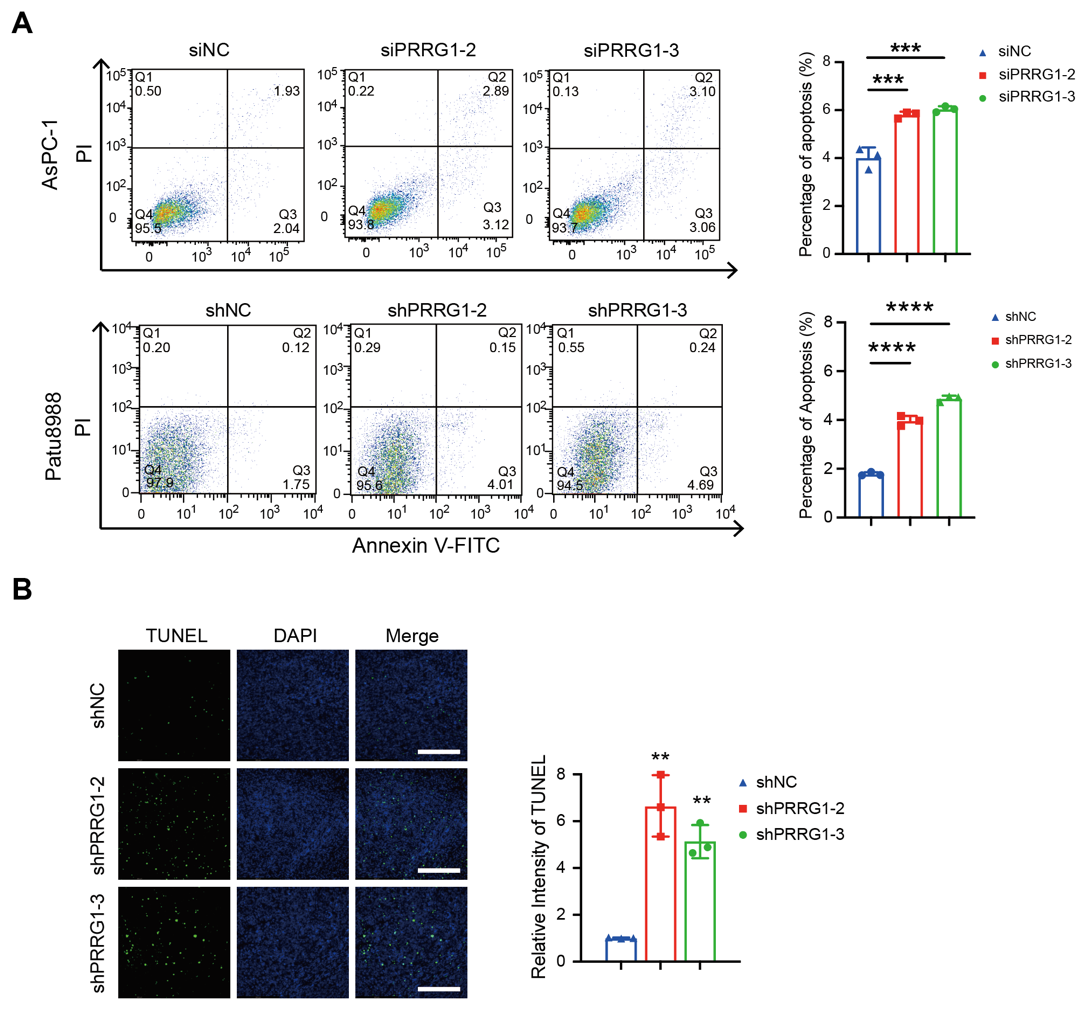


Supplementary Figure 2. (A) Analysis of apoptosis induced by serum-free starvation in PRRG1-knockdown and control cells. The percentages of Annexin V-positive apoptotic cells were analyzed using 1-way ANOVA with Dunnett’s post hoc test (n = 3 for each group). (B) TUNEL labeling of apoptotic cells (green) in subcutaneous tumors derived from PRRG1-knockdown or control Patu8988 cells (n = 3 for each group). Scale bar: 250 μm. Statistical analysis of TUNEL-positive apoptotic cells were shown on the right panel. Mean ± SD, 1-way ANOVA with Dunnett’s post hoc test. **P < 0.01. ***P < 0.001. ****P < 0.0001.


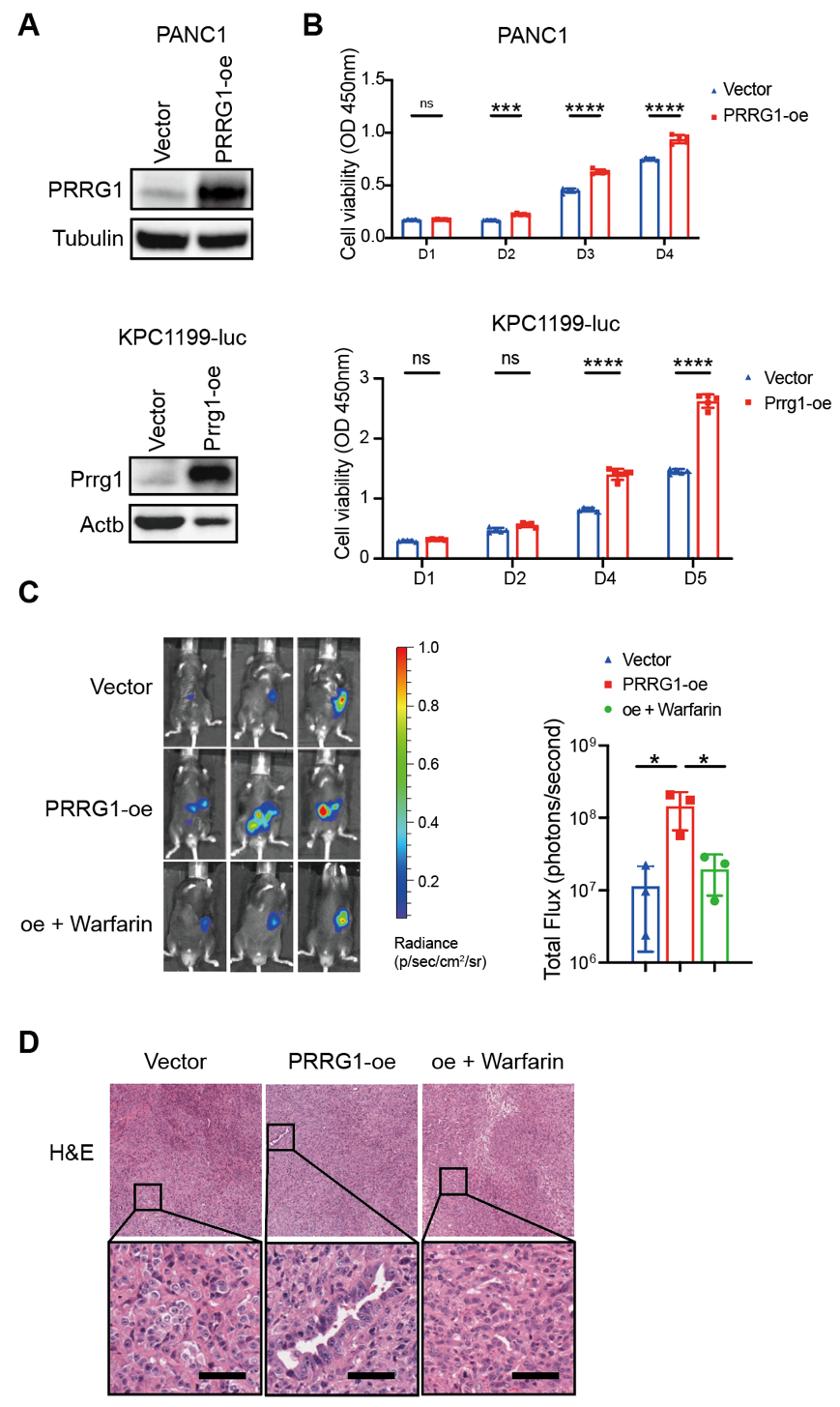


Supplementary Figure 3. (A) Overexpression of PRRG1-Flag in PANC1 and Prrg1-Flag in KPC1199^luc^ cells. (B) Cell proliferation of PANC1 cells with PRRG1-Flag overexpression and KPC1199^luc^ cells with Prrg1-Flag overexpression. (C) Growth of in vivo orthotopic tumors, derived from vector and Prrg1-overexpression KPC1199^luc^ cells, measured by an in vivo imaging system. Statistical analysis of the total Flux (photons/second) was shown besides (n = 3). (D) Representative images of H&E staining of the orthotopic tumors derived from vector-control and Prrg1-overexpression KPC1199^luc^ cells, with or without warfarin treatment. Scale bar: 50 μm. Mean ± SD, 1-way ANOVA with Dunnett’s post hoc test or 2-way ANOVA with Bonferroni’s post hoc tests. *P < 0.05. ***P < 0.001. ****P < 0.0001.


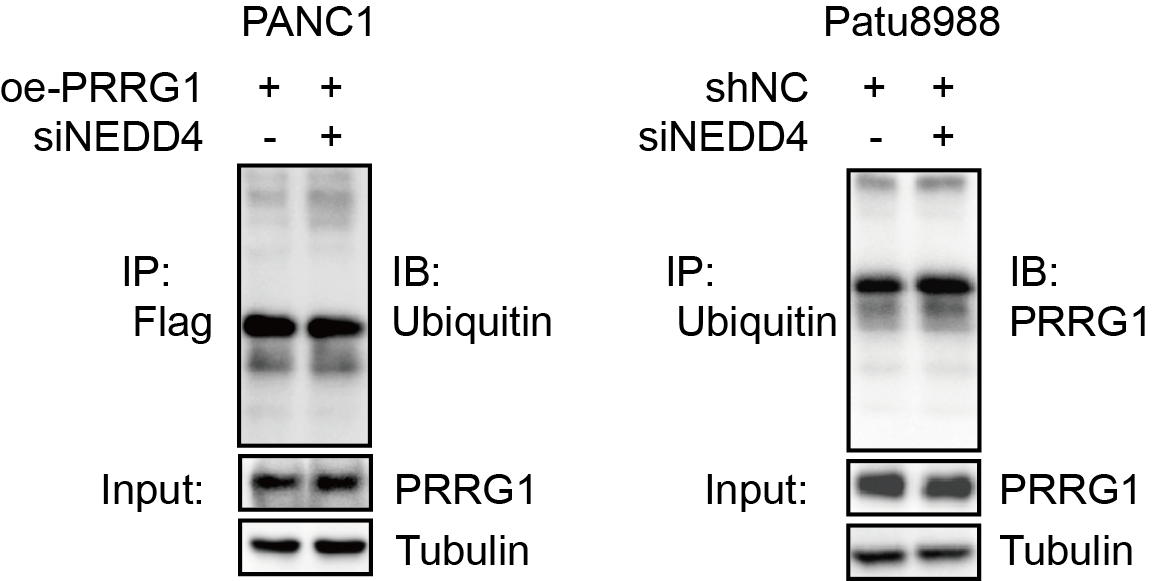


Supplementary Figure 4. PRRG1 ubiquitination was not apparently altered by NEDD4 knockdown. Left, immunoprecipitation of the Flag tag from PRRG1-overexpression PANC1 cells with or without siRNA-mediated NEDD4 knockdown, followed by western blotting with the antibody against ubiquitin. Right, immunoprecipitation of ubiquitin from Patu8988 cells with or without NEDD4 knockdown, followed by western blotting with an antibody against PRRG1. Images are representative from two independent experiments.


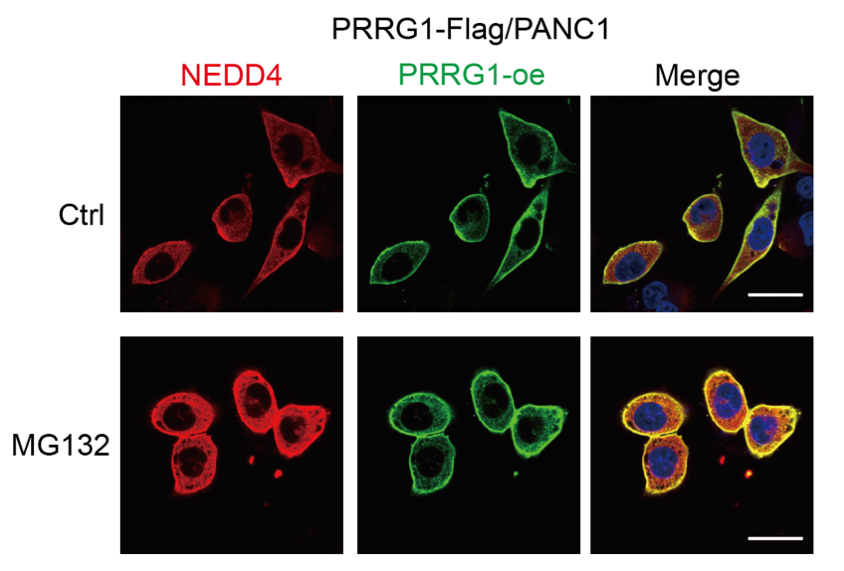


Supplementary Figure 5. Representative images of co-immunofluorescence of NEDD4 (red) and PRRG1-Flag (green) in PANC1 cells with PRRG1-flag overexpression. Images are representative from three different field of two independent experiments. Scale bar: 25 μm.


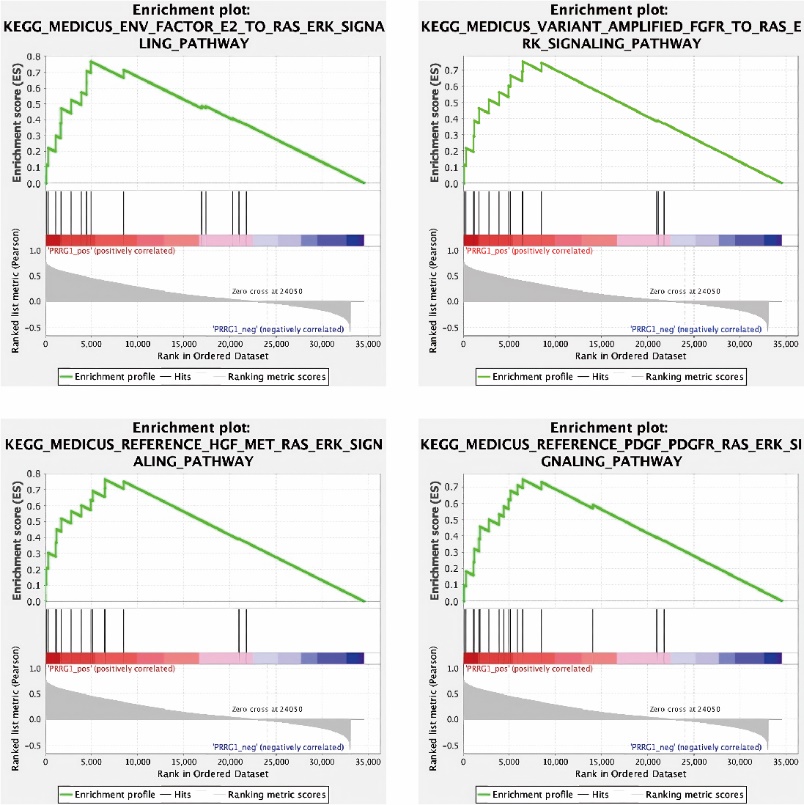


Supplementary Figure 6. Gene Set Enrichment Analysis (GSEA) analysis of co-expressed genes of PRRG1 in TCGA-PAAD database exhibited a close correlation with Ras signaling.


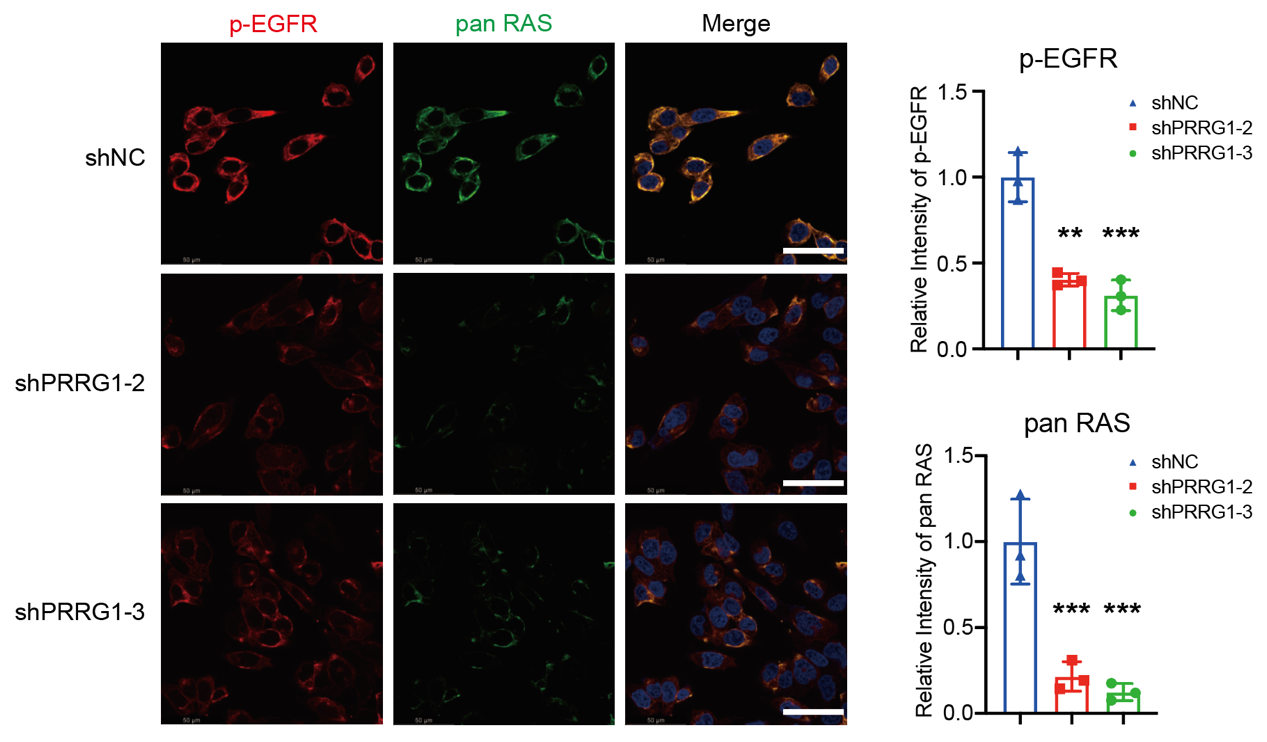


Supplementary Figure 7. Co-immunofluorescence staining of p-EGFR and pan RAS in control and PRRG1-knockdown Patu8988 cells. Mean ± SD, 1-way ANOVA with Dunnett’s post hoc test (n=3 for each group). **P < 0.01. ***P < 0.001. Scale bar: 50 μm.

﻿**Supplementary Tables**

**Supplementary Table 1.** 44 shared genes obtained from the intersection of commonly upregulated genes in three GEO datasets, prognosis-related genes and KRAS co-expressed genes in TCGA-PAAD.

| ﻿Gene Symbol | Gene ID | Gene Name |
| --- | --- | --- |
| *YAP1* | 10413 | Yes1 associated transcriptional regulator |
| *TES* | 26136 | testin LIM domain protein |
| *SAMD9* | 54809 | sterile alpha motif domain containing 9 |
| *RPE* | 6120 | ribulose-5-phosphate-3-epimerase |
| *LAMA3* | 3909 | laminin subunit alpha 3 |
| *KIF14* | 9928 | kinesin family member 14 |
| *LAMC2* | 3918 | laminin subunit gamma 2 |
| *SGMS2* | 166929 | sphingomyelin synthase 2 |
| *ASAP2* | 8853 | ArfGAP with SH3 domain, ankyrin repeat and PH domain 2 |
| *IL1RAP* | 3556 | interleukin 1 receptor accessory protein |
| *IQGAP1* | 8826 | IQ motif containing GTPase activating protein 1 |
| *SLC5A3* | 6526 | solute carrier family 5 member 3 |
| *EPS8* | 2059 | EGFR pathway substrate 8, signaling adaptor |
| *EFNB2* | 1948 | ephrin B2 |
| *ARHGAP42* | 143872 | Rho GTPase activating protein 42 |
| *LDHA* | 3939 | lactate dehydrogenase A |
| *FGD6* | 55785 | FYVE, RhoGEF and PH domain containing 6 |
| *PERP* | 64065 | p53 apoptosis effector related to PMP22 |
| *LIPH* | 200879 | lipase H |
| *PRRG1* | 5638 | proline rich and Gla domain 1 |
| *ITGB6* | 3694 | integrin subunit beta 6 |
| *ECT2* | 1894 | epithelial cell transforming 2 |
| *NCK1* | 4690 | NCK adaptor protein 1 |
| *PRKCI* | 5584 | protein kinase C iota |
| *TMOD3* | 29766 | tropomodulin 3 |
| *ITGA2* | 3673 | integrin subunit alpha 2 |
| *MYOF* | 26509 | myoferlin |
| *SLC39A10* | 57181 | solute carrier family 39 member 10 |
| *ADAM10* | 102 | ADAM metallopeptidase domain 10 |
| *LMO7* | 4008 | LIM domain 7 |
| *PTPN12* | 5782 | protein tyrosine phosphatase non-receptor type 12 |
| *SERPINB5* | 5268 | serpin family B member 5 |
| *CTTNBP2NL* | 55917 | CTTNBP2 N-terminal like |
| *ASPM* | 259266 | assembly factor for spindle microtubules |
| *MYO1E* | 4643 | myosin IE |
| *ANLN* | 54443 | anillin, actin binding protein |
| *INPP4B* | 8821 | inositol polyphosphate-4-phosphatase type II B |
| *GPRC5A* | 9052 | G protein-coupled receptor class C group 5 member A |
| *CDCP1* | 64866 | CUB domain containing protein 1 |
| *B3GNT5* | 84002 | UDP-GlcNAc:betaGal beta-1,3-N-acetylglucosaminyltransferase 5 |
| *REEP3* | 221035 | receptor accessory protein 3 |
| *ADAM9* | 8754 | ADAM metallopeptidase domain 9 |
| *MET* | 4233 | MET proto-oncogene, receptor tyrosine kinase |
| *B4GALT5* | 9334 | beta-1,4-galactosyltransferase 5 |

**Supplementary Table 2.** ﻿Correlation of PRRG1 and pathological characteristics of PDAC patients.

|  |  | **Expression of PRRG1** | |  |
| --- | --- | --- | --- | --- |
| **Clinicopathological parameters** | **Total** | **Low** | **High** | ***P* value** |
| **Age，y** |  |  |  | .156 |
| ≤65 | 33 | 11 | 22 |  |
| >65 | 51 | 25 | 26 |  |
| **Gender** |  |  |  | .897 |
| Male | 52 | 22 | 30 |  |
| Female | 32 | 14 | 18 |  |
| **Tumor location** |  |  |  | .393 |
| 1 | 54 | 25 | 29 |  |
| 2 | 30 | 11 | 19 |  |
| **TNM stage** |  |  |  | .513 |
| I | 38 | 18 | 20 |  |
| II | 39 | 15 | 24 |  |
| III | 3 | 1 | 2 |  |
| IV | 4 | 2 | 2 |  |
| **Lymphatic metastasis** |  |  |  | **.004** |
| Absent | 53 | 29 | 24 |  |
| Present | 31 | 7 | 24 |  |
| **Vascular metastasis** |  |  |  | .794 |
| Absent | 71 | 30 | 41 |  |
| Present | 13 | 6 | 7 |  |
| **Intra-abdominal metastasis** |  |  |  |  |
| Absent | 82 | 35 | 47 | 1.000 |
| Present | 2 | 1 | 1 |  |

**Supplementary Table 3.** ﻿siRNA Sequences.

| ﻿siRNA | ﻿Fragment | ﻿ Sequence (5’- 3’) |
| --- | --- | --- |
| *si-PRRG1-1* | ﻿Sense RNA  ﻿Antisense RNA | GAGGAGUGAAACAGAACCUCAdTdT  UGAGGUUCUGUUUCACUCCUCdTdT |
| *si-PRRG1-2* | ﻿Sense RNA  ﻿Antisense RNA | AGGAAGUGACUGGUUUCAGUUdTdT  AACUGAAACCAGUCACUUCCUdTdT |
| *si-PRRG1-3* | ﻿Sense RNA  ﻿Antisense RNA | UCCGUUAAUCUUUGGCCUCUUdTdT  AAGAGGCCAAAGAUUAACGGAdTdT |
| *si-NEDD4* | Sense RNA  ﻿Antisense RNA | UAGAGCCUGGCUGGGUUGUUUUGdTdT  CAAAACAACCCAGCCAGGCUCUAdTdT |

**Supplementary Table 4.** ﻿shRNA Sequences.

| ﻿shRNA | ﻿ Sequence |
| --- | --- |
| *shNC* | CCGGCCTAAGGTTAAGTCGCCCTCGCTCGAGCGAGGGCGACTTAACCTTAGGTTTTTTG |
| *sh-PRRG1-1* | CCGGGAAATAAGACAGGGCAACATTCTCGAGAATGTTGCCCTGTCTTATTTCTTTTTTG |
| *sh-PRRG1-2* | CCGGACGCTACCCAAGAGCTAATTTCAAGAGAATTAGCTCTTGGGTAGCGTTTTTTTG |
| *sh-PRRG1-3* | CCGGGCGTGAGTGCAAAGAAGAATTCAAGAGATTCTTCTTTGCACTCACGCTTTTTTG |
